# Wood-feeding termite gut symbionts as an obscure yet promising source of novel manganese peroxidase-producing oleaginous yeasts intended for azo dye decolorization and biodiesel production

**DOI:** 10.1186/s13068-021-02080-z

**Published:** 2021-12-04

**Authors:** Rania Al-Tohamy, Jianzhong Sun, Maha A. Khalil, Michael Kornaros, Sameh Samir Ali

**Affiliations:** 1 School of the Environment and Safety Engineering, Biofuels Institute, Jiangsu University, Xuefu Road 301, Zhenjiang, 212013 China; 2 Department of Biology, College of Science, Taif University, P.O. Box 11099, Taif, 21944 Saudi Arabia; 3 Laboratory of Biochemical Engineering & Environmental Technology (LBEET), Department of Chemical Engineering, University of Patras, University Campus, 1 Karatheodori Str, 26504 Patras, Greece; 4 INVALOR: Research Infrastructure for Waste Valorization and Sustainable Management, University Campus, 26504 Patras, Greece; 5 Botany Department, Faculty of Science, Tanta University, Tanta, 31527 Egypt

**Keywords:** *Meyerozyma caribbica*, Oleaginous yeasts, Azo dyes, Manganese peroxidases, Lignocellulose degradation inhibitors, Wood-feeding termite gut symbionts

## Abstract

**Background:**

The ability of oxidative enzyme-producing micro-organisms to efficiently valorize organic pollutants is critical in this context. Yeasts are promising enzyme producers with potential applications in waste management, while lipid accumulation offers significant bioenergy production opportunities. The aim of this study was to explore manganese peroxidase-producing oleaginous yeasts inhabiting the guts of wood-feeding termites for azo dye decolorization, tolerating lignocellulose degradation inhibitors, and biodiesel production.

**Results:**

Out of 38 yeast isolates screened from wood-feeding termite gut symbionts, nine isolates exhibited high levels of extracellular manganese peroxidase (MnP) activity ranged between 23 and 27 U/mL after 5 days of incubation in an optimal substrate. Of these MnP-producing yeasts, four strains had lipid accumulation greater than 20% (oleaginous nature), with *Meyerozyma caribbica* SSA1654 having the highest lipid content (47.25%, w/w). In terms of tolerance to lignocellulose degradation inhibitors, the four MnP-producing oleaginous yeast strains could grow in the presence of furfural, 5-hydroxymethyl furfural, acetic acid, vanillin, and formic acid in the tested range. *M. caribbica* SSA1654 showed the highest tolerance to furfural (1.0 g/L), 5-hydroxymethyl furfural (2.5 g/L) and vanillin (2.0 g/L). Furthermore, *M. caribbica* SSA1654 could grow in the presence of 2.5 g/L acetic acid but grew moderately. Furfural and formic acid had a significant inhibitory effect on lipid accumulation by *M. caribbica* SSA1654, compared to the other lignocellulose degradation inhibitors tested. On the other hand, a new MnP-producing oleaginous yeast consortium designated as NYC-1 was constructed. This consortium demonstrated effective decolorization of all individual azo dyes tested within 24 h, up to a dye concentration of 250 mg/L. The NYC-1 consortium's decolorization performance against Acid Orange 7 (AO7) was investigated under the influence of several parameters, such as temperature, pH, salt concentration, and co-substrates (e.g., carbon, nitrogen, or agricultural wastes). The main physicochemical properties of biodiesel produced by AO7-degraded NYC-1 consortium were estimated and the results were compared to those obtained from international standards.

**Conclusion:**

The findings of this study open up a new avenue for using peroxidase-producing oleaginous yeasts inhabiting wood-feeding termite gut symbionts, which hold great promise for the remediation of recalcitrant azo dye wastewater and lignocellulosic biomass for biofuel production.

**Graphical Abstract:**

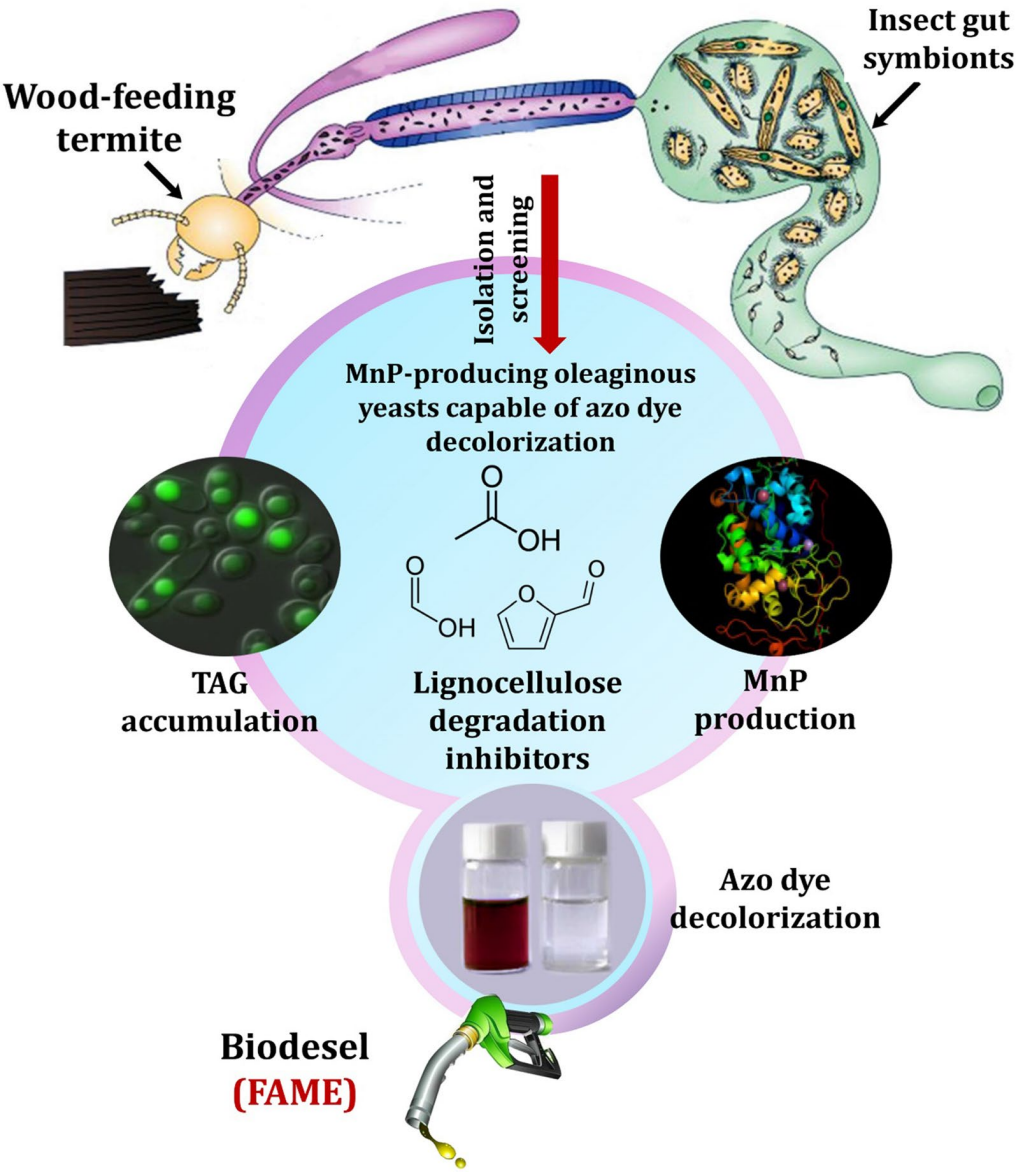

**Supplementary Information:**

The online version contains supplementary material available at 10.1186/s13068-021-02080-z.

## Background

Lignocellulosic biomass is a critical raw resource for a number of existing industries, such as forestry, pulp and paper, and growing second-generation biofuel manufacturing [1–7]. Among lignocellulosic components, lignin is the major and most recalcitrant aromatic macromonomer compound responsible for its strength [8, 9]. So far, the only source of energy that has been known to be easily accessible for both biomass-based and green energy production as well as the bio-based manufacture of aromatics and new polymers is lignin [10, 11]. The amorphous, heteropolymeric lignin, which comprises phenylpropanoid units, may be deconstructed into low molecular weight and monomeric aromatic compounds that serve as the building block for high-value products [12, 13]. Recent economic analyses have revealed that lignin valorization might produce at least ten times more value than burning it for energy production [14–17]. However, the cost of delignification processing, required in many industrial systems, has proven difficult and expensive. To adequately address these difficulties, microbial degradation was introduced. Wood-rotting basidiomycetes have been widely investigated for their inherent and efficient delignification activity [18–20]. For large-scale lignin biodegradation, delignifying enzymes such as lignin peroxidase (LiP; EC 1.11.1.14), manganese peroxidase (MnP; EC 1:11:1:13), and laccase (Lac; EC 1.10.3.2) were introduced. MnP is the major enzyme class in lignin biodegradation by white-rot fungi [21, 22]. For the pulp and paper industries, as well as for the management of xenobiotic pollutants, such as textile dyes, using lignin-degrading organisms and their constituent enzymes has become appealing [23–26].

Large amounts of potentially hazardous pollutants have been released into the biosphere as a result of global industrialization [27, 28]. Cleaning up the environment by removing dangerous chemicals from textile effluents is a critical and difficult subject that necessitates a variety of ways to find long-term solutions [29]. Among the numerous types of dyes used in the textile processing industry, azo-dyes are widely employed, and they account for over 70% of the dyestuff market [30]. Physical and chemical methods for treating azo-dyes have been used, however, resulting in secondary contamination from the release of hazardous by-products [24, 31]. As a result of its safety, efficiency, and capacity to change dangerous chemicals into less toxic compounds, microbial treatment of azo-dyes has grown in popularity. The ability of dye-decolorizing/ligninolytic microbes to degrade resistant xenobiotics, such as lignin-mimicking synthetic dyes, may represent a significant opportunity for the biodegradation of such recalcitrant xenobiotics. Recent research has extensively looked at the bioremediation of azo dyes by bacteria and fungus [32, 33]. Still, the records on yeasts' success against difficult azo dyes are confined to only a few documents [34–36]. Yeasts possess higher resistance to harsh conditions, such as severe pH, osmolality, and temperature. At the same time, they have excellent biodegradation power by creating numerous enzymes that target certain harmful contaminants [37].

Biodiesel, also known as fatty acid methyl ester (FAME) is a viable alternative resource that can efficiently replace petroleum-based diesel in terms of new renewable energy sources [38, 39]. To this end, single cell oil (SCO), also known as a microbial lipid, has received significant attention due to its renewability, sustainability, and extensive biotechnological use. At the same time, one of the most critical criteria of SCO quality is the fatty acid composition and its similarity with vegetable oil [40]. The major lipid type of SCO is triacylglycerides (TAG), which are composed of long-chain fatty acids. Several microbial species, including fungi, yeasts, bacteria, and microalgae, can produce and accumulate enormous amounts of lipids under particular conditions [41]. Yeasts are classified as oleaginous when their ability to accumulate neutral lipids, particularly TAG, exceeds 20% (w/w) on a dry weight basis [42]. Thus, lipid-accumulating yeast species from the yeast genera *Yarrowia*, *Candida*, *Cryptococcus*, *Rhodotorula,* and *Lipomyces* have received increased attention for biofuel production [43, 44]. Concurrently, the ability of oleaginous yeasts to metabolize aromatic substances may boost lipid yields, since the ortho-cleavage pathway of aromatic metabolism creates acetyl CoA and pyruvic acid, both of which are precursors for fatty acid biosynthesis [45]. Furthermore, yeasts have been identified as prospective enzyme manufacturers, producing xylanases, cellulases, proteases, and lipases, all of which are widely used in bioenergy and waste management [35, 43, 46].

Several studies have recently focused on the gut digestome of xylophagous insects, such as wood-feeding termites (WFTs), to find and understand their specific symbiont activities and potential biotechnological applications. However, the highly specialized gut symbionts of WFTs, notably yeast symbionts, are still little understood. To our knowledge, the biotechnological potential of some promising yeast species derived from *Reticulitermes chinensis* and *Coptotermes formosanus* for degrading recalcitrant xenobiotics such as textile azo dyes has been emphasized for the first time [32, 47]. Furthermore, the potential of oleaginous yeasts inhabiting WFTs to degrade lignin and lignin-like dyes into metabolites usable for biodiesel production is almost unexplored. Under this scope, this study aims at exploring MnP-producing oleaginous yeasts capable of decolorizing textile azo dyes and tolerating lignocellulose degradation inhibitors. As a result, peroxidase-producing oleaginous yeasts may be a potential candidate for bioremediating textile wastewater, valorizing lignocellulose, and producing biodiesel.

## Results

### Screening of peroxidase- and lipid-accumulating yeasts inhabiting WFT guts

In this study, *C. formosanus* and *R. chinenesis* were used to isolate and screen MnP- and lipid-accumulating yeasts intended for decolorizing textile-derived azo dyes, tolerating lignocellulose degradation inhibitors, and producing biodiesel (FAME). Based on clear zone development on yeast extract peptone dextrose (YEPD) supplemented with o-Dianisidine dihydrochloride as a precursor of numerous azo dyes and a peroxidase substrate, 38 isolated yeasts yielded positive findings for MnP production. Furthermore, the emergence of a reddish-brown color between 31 yeast colonies after plate treatment with H_2_O_2_ was confirmed, indicating the production of the MnP enzyme. Nine isolates designated as PPY-3, PPY-4, PPY-11, PPY-13, PPY-17, PPY-22, PPY-27, PPY-29, and PPY-35 exhibited high levels of extracellular MnP activity after 5 days of incubation in an optimal substrate. This activity ranged between 23 and 27 U/mL. On the other hand, 14 of 22 MnP-producing yeast isolates were qualitatively stained blue for TAG accumulation. Fluorometric analysis with Nile red staining, however, indicated seven yeast isolates capable of MnP production, namely, PPY-4, PPY-11, PPY-18, PPY-22, PPY-27, PPY-32, and PPY-35, with substantial yellow-gold colouring.

Table 1 depicts the molecular identification and relationship of these seven MnP-producing/TAG-accumulating yeasts to their closest phylogenetic relatives based on BLAST comparisons to the GeneBank database. The four yeast isolates PPY-11, PPY-22, PPY-4, and PPY-35 were identified as *Meyerozyma caribbica* strain SSA1654, *Meyerozyma guilliermondii* strain SSA1547, *Candida stauntonica* strain SSA1653, and *Debaryomyces hansenii* strain SSA1502, respectively, belonging to the Ascomycota phylum. The remaining three yeast isolates, PPY-32, PPY-27, and PPY-18, belonged to the Basidiomycota phylum and were identified as *Fellozyma inositophila* strain SSA1579, *Sterigmatomyces halophilus* strain SSA1655, and *Vanrija humicola* strain SSA1514, respectively.

**Table 1:** Identification of MnP-producing/TAG-accumulating yeasts based on BLAST comparisons to the GeneBank database

Isolate code	Yeast species	Strain	Accession no.	Closest relative [accession no.]	Sequence identity (%)
PPY-4	[*Candida*] *stauntonica*	SSA1653	KY172950	*Candida stauntonica* strain ATCC MYA-4699 [JQ812698]	95.40
PPY-11	*Meyerozyma caribbica* (former *Candida fermentati*)	SSA1654	KY172951	*Meyerozyma caribbica* strain LZ-12 [JQ686909]	99.30
PPY-18	*Vanrija humicola* (former *Cryptococcus humicola*)	SSA1514	KX791400	*Vanrija humicola* strain SSA1520 [KX791406]	99.34
PPY-22	*Meyerozyma guilliermondii* (former *Pichia guilliermondii*)	SSA1547	KX907633	*Meyerozyma guilliermondii* strain ML4 [MK907983]	100.00
PPY-27	*Sterigmatomyces halophilus*	SSA1655	KY172952	*Sterigmatomyces halophilus* strain KU-79 [MG815870]	99.67
PPY-32	*Fellozyma inositophila*	SSA1579	KX791364	*Fellozyma inositophila* strain CBS 7310 [AF189987]	95.44
PPY-35	*Debaryomyces hansenii*	SSA1502	KX791388	*Debaryomyces hansenii* strain LL2 [EU131182]	99.49

**Fig. 1:**
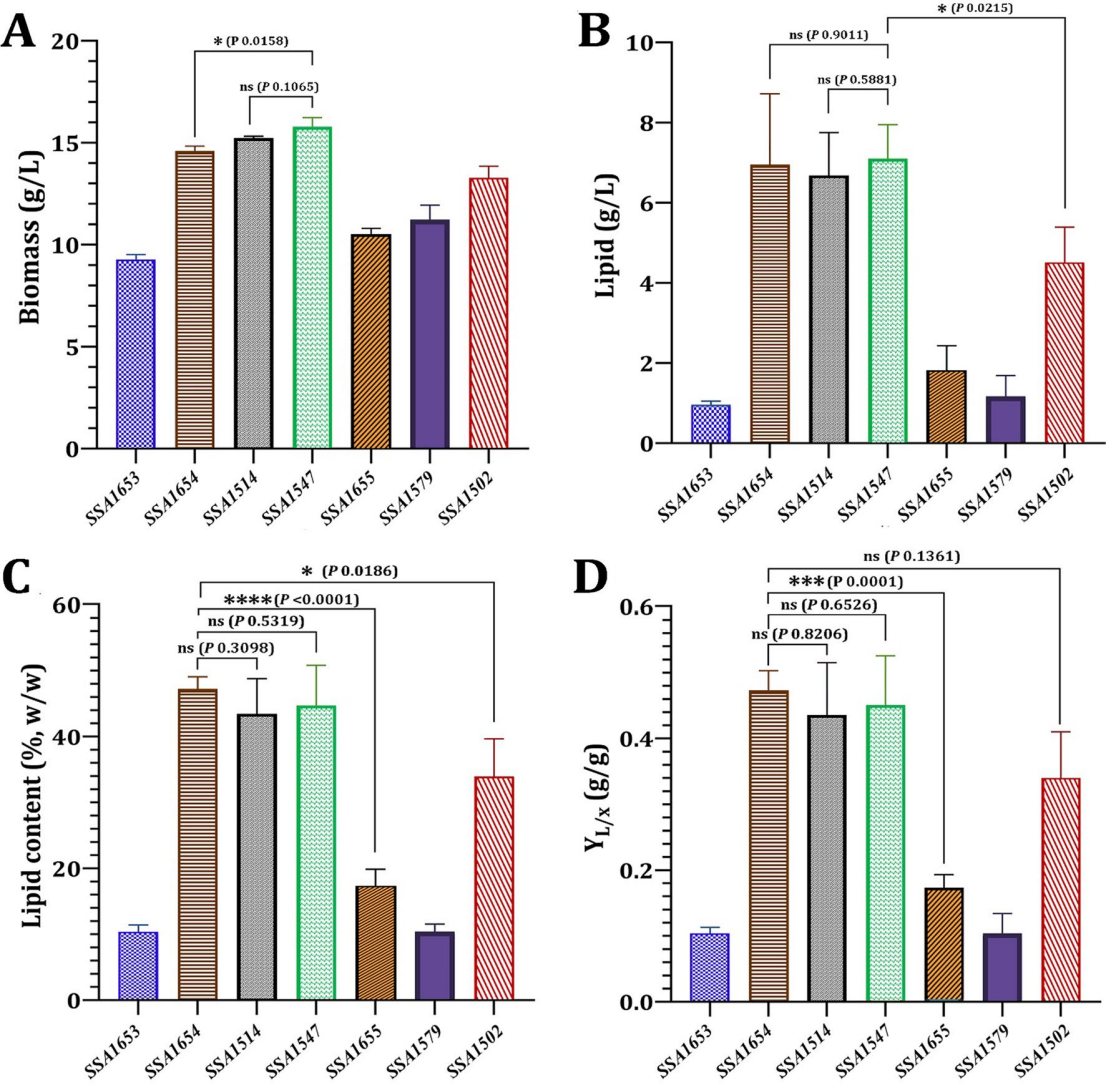
Lipid accumulation profile of seven MnP-producing yeast strains in terms of biomass (**A**), lipid concentration (**B**), lipid content (**C**), and lipid yield (**D**). The oleaginous and non-oleaginous MnP-yeast strains used in this study: *Candida stauntonica* strain SSA1653, *Meyerozyma caribbica* strain SSA1654, *Vanrija humicola* strain SSA1514, *Meyerozyma guilliermondii* strain SSA1547, *Sterigmatomyces halophilus* strain SSA1655, *Fellozyma inositophila* strain SSA1579, *Debaryomyces hansenii* strain SSA1502. **Y**_**L/x**_ is the lipid yield (g) per gram of total biomass produced. Values are the mean of three independent replicates, with error bars indicating the standard deviation

**Fig. 2:**
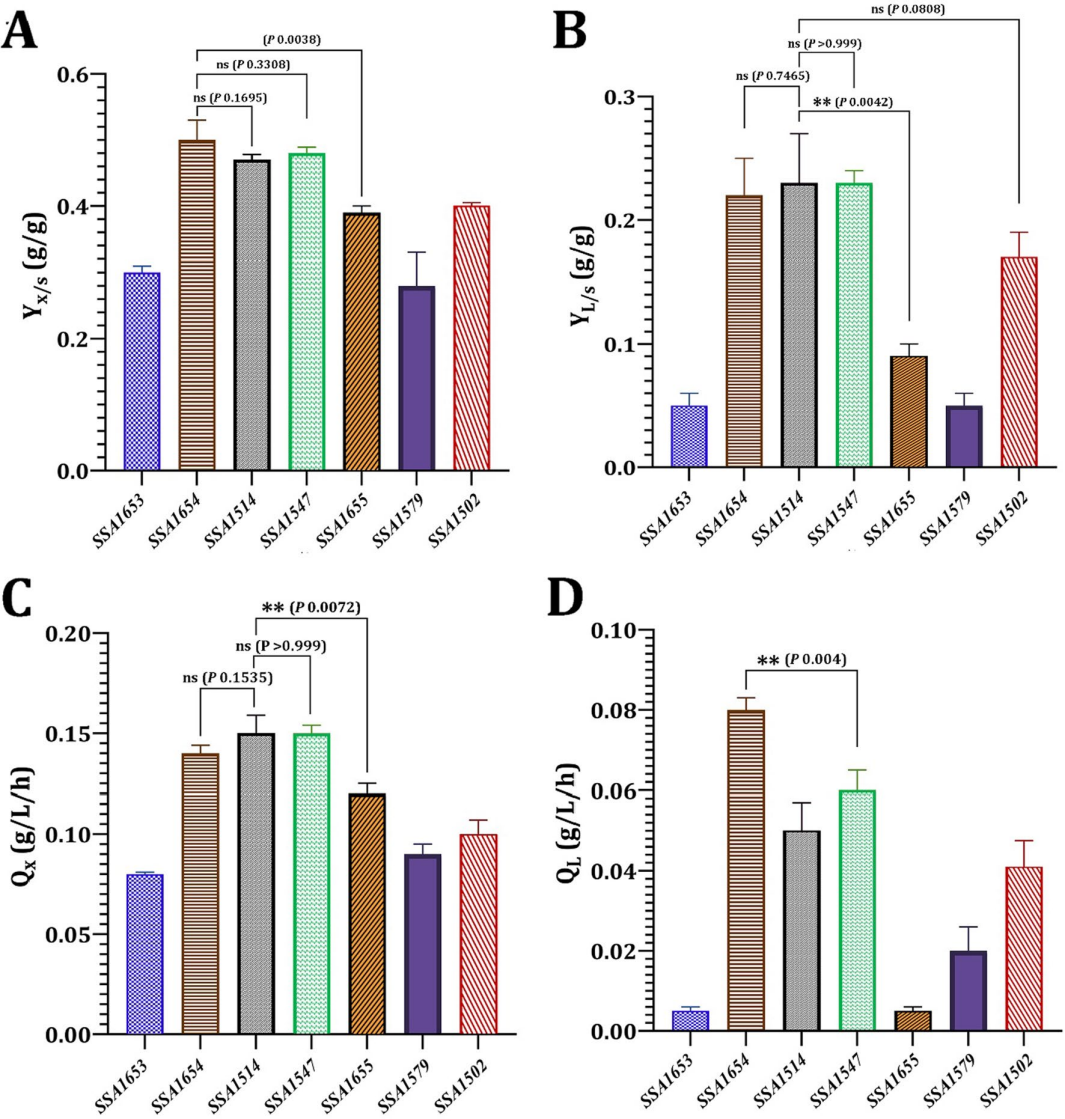
Lipid production by seven MnP-producing yeast strains in terms of biomass yield (**A**), lipid yield (**B**), biomass productivity (**C**), and lipid productivity (**D**). The oleaginous and non-oleaginous MnP–yeast strains used in this study: *Candida stauntonica* strain SSA1653, *Meyerozyma caribbica* strain SSA1654, *Vanrija humicola* strain SSA1514, *Meyerozyma guilliermondii* strain SSA1547, *Sterigmatomyces halophilus* strain SSA1655, *Fellozyma inositophila* strain SSA1579, *Debaryomyces hansenii* strain SSA1502. **Y**_**x/s**_ is the yield (g) of biomass produced per gram of glucose consumed. **Y**_**L/s**_ is the lipid yield (g) produced per gram of glucose consumed. Q_x_ is the volumetric productivity of biomass. Q_L_ is the volumetric productivity of lipid. Values are the mean of three independent replicates, with error bars indicating the standard deviation

The lipid profiles of the selected seven yeast strains were evaluated after 5 days of growth at 28 °C in the presence of glucose and under nitrogen limitation (Figs. 1 and 2). *M. guilliermondii* SSA1547 has the highest biomass value (15.78 ± 0.45 g/L). It revealed a non-significant difference (*p* 0.1065) with biomass produced by *V. humicola* SSA1514, but a significant difference (*p* 0.0158) with biomass produced by the closest yeast strain, *M. caribbica* SSA1654 (Fig. 1a). In terms of lipid yield (Fig. 1b), there was no significant difference between *M. guilliermondii* SSA1547 (7.10 ± 0.85 g/L), *V. humicola* SSA1514 (6.62 ± 1.13 g/L), and *M. caribbica* SSA1654 (6.95 ± 1.77 g/L). It differed significantly (*p* 0.0215) from the lipid produced by *D. hansenii* SSA1502 (4.51 ± 0.88 g/L) (Fig. 1b). The lipid content was subsequently quantified to verify the yeasts’ oleaginous nature (Fig. 1c). *S. halophilus* SSA1655, *F. inositophila* SSA1579, and *C. stauntonica* SSA1653 accumulated less than 20% lipids per dry biomass when lipid content was determined (Fig. 1c). In contrast, four yeast strains had lipid accumulation greater than 20%, with *M. caribbica* SSA1654 having the highest lipid content (47.25 ± 1.84%, w/w), followed by *M. guilliermondii* SSA1547 (44.74 ± 6.09%, w/w), *V. humicola* SSA1514 (43.46 ± 5.34%, w/w), and *D. hansenii* SSA1502 (33.96 ± 5.72%, w/w) (Fig. 1c). As a result, *S. halophilus* SSA1655, *F. inositophila* SSA1579, and *C. stauntonica* SSA1653 were found to be non-oleaginous yeasts, whereas *M. caribbica* SSA1654, *M. guilliermondii* SSA1547, *V. humicola* SSA1514, and *D. hansenii* SSA1502 were found to be oleaginous yeast strains. Although *M. caribbica* SSA1654 had no significant difference in lipid content from *M. guilliermondii* SSA1547 (*p* 0.5319) or *V. humicola* SSA1514 (*p* 0.3098), the latter three strains had significantly higher lipid content than *S. halophilus* SSA1655 (*p* < 0.0001) and *D. hansenii* (*p* 0.0186). Y_L/x_, the lipid yield per gram of total biomass produced, is shown in Fig. 1d. When compared to oleaginous and non-oleaginous yeast strains examined, *M. caribbica* SSA1654 had the highest Y_L/x_ (0.340 ± 0.07 g/g). Furthermore, *M. caribbica* SSA1654 exhibited no significant difference (*p* 0.8206) from *V. humicola* SSA1514 in terms of Y_L/x_. When compared to *M. guilliermondii* SSA1547 (*p* 0.6526) and *D. hansenii* SSA1502 (*p* 0.1361), the latter two strains exhibited no significant difference in Y_L/x_. *M. caribbica* SSA1654, *V. humicola* SSA1514, and *M. guilliermondii* SSA1547, on the other hand, revealed a substantially greater Y_L/x_ than *S. halophilus* SSA1655 (*p* 0.0001) (Fig. 1d).

Y_x/s_, the yield of biomass produced per gram of glucose consumed, is shown in Fig. 2a. *M.* *caribbica* SSA1654 had the highest Y_x/s_ (0.50 ± 0.03 g/g), with a considerably higher Y_x/s_ (*p* 0.0038) than *S. halophilus* SSA1655. However, no significant difference in Y_x/s_ was determined between *M. caribbica* SSA1654 and *V. humicola* SSA1514 (*p* 0.1695) or *M. guilliermondii* SSA1547 (*p* 0.3308). Y_L/s_, the lipid yield produced per gram of glucose consumed, is shown in Fig. 2b. In terms of Y_L/s_, *V. humicola* SSA1514 had the highest Y_L/s_ value of 0.23 ± 0.04 g/g, with a significantly higher Y_L/s_ than *S. halophilus* SSA1655 (0.09 ± 0.01 g/g; *p* 0.0042). There was no significant difference in volumetric productivity of biomass (Qx) between *V. humicola* SSA1514 (0.15 ± 0.009 g/L/h) and *M. caribbica* SSA1654 (*p* 0.1535) or *M. guilliermondii* SSA1547 (*p* > 0.99). *V. humicola* SSA1514, on the other hand, had a significantly higher Qx than *S. halophilus* SSA1655 (*p* < 0.0072) (Fig. 2c). *M. caribbica* SSA1654 had the highest volumetric production of lipids (Q_L_; 0.08 ± 0.003 g/L/h), as shown in Fig. 2d, with a significant difference from *M. guilliermondii* SSA1547 (*p* 0.004). Table 2 compares lipid production of the oleaginous strains to that of other oleaginous yeasts reported in the literature.

**Table 2:** Lipid production by MnP-producing oleaginous yeast strains using shaking flask in lipid production medium containing glucose and o-Dianisidine dihydrochloride (as a precursor of many azo dyes and a peroxidase substrate)

Yeast	Biomass (g/L)	Lipid (g/L)	Lipid content (%, w/w)	Carbon source	References
*Candida viswanathii* Y-E4	13.6	3.4	25.3	Glucose	Ayadi et al. [58]
*Cystobasidium oligophagum* JRC1	12.34	4.9	39.4	Glucose	Vyas and Chhabra [7]
*Yarowia lipolytica* MUCL28849	30.8	12.4	40.7	Glucose + acetic acid	Fontanille et al. [59]
*Cryptococcus vishniaccii*	13.6	5.5	40.4	Glucose	Deeba et al. [60]
*Rhodosporidium kratochvilovae* HIMPA1	14.5	6.2	41.9	Glucose	Patel et al. [61]
*Meyerozyma guilliermondii* strain SSA1547	15.8	7.1	44.7	Glucose	This study
*Meyerozyma caribbica* strain SSA1654	14.8	7.0	47.3	Glucose	This study
*Vanrija humicola* strain SSA1514	15.2	6.7	43.5	Glucose	This study
*Debaryomyces hansenii* strain SSA1502	13.3	4.5	34.0	Glucose	This study

### Profiles of oleaginous and non-oleaginous yeast fermentation

**Fig. 3:**
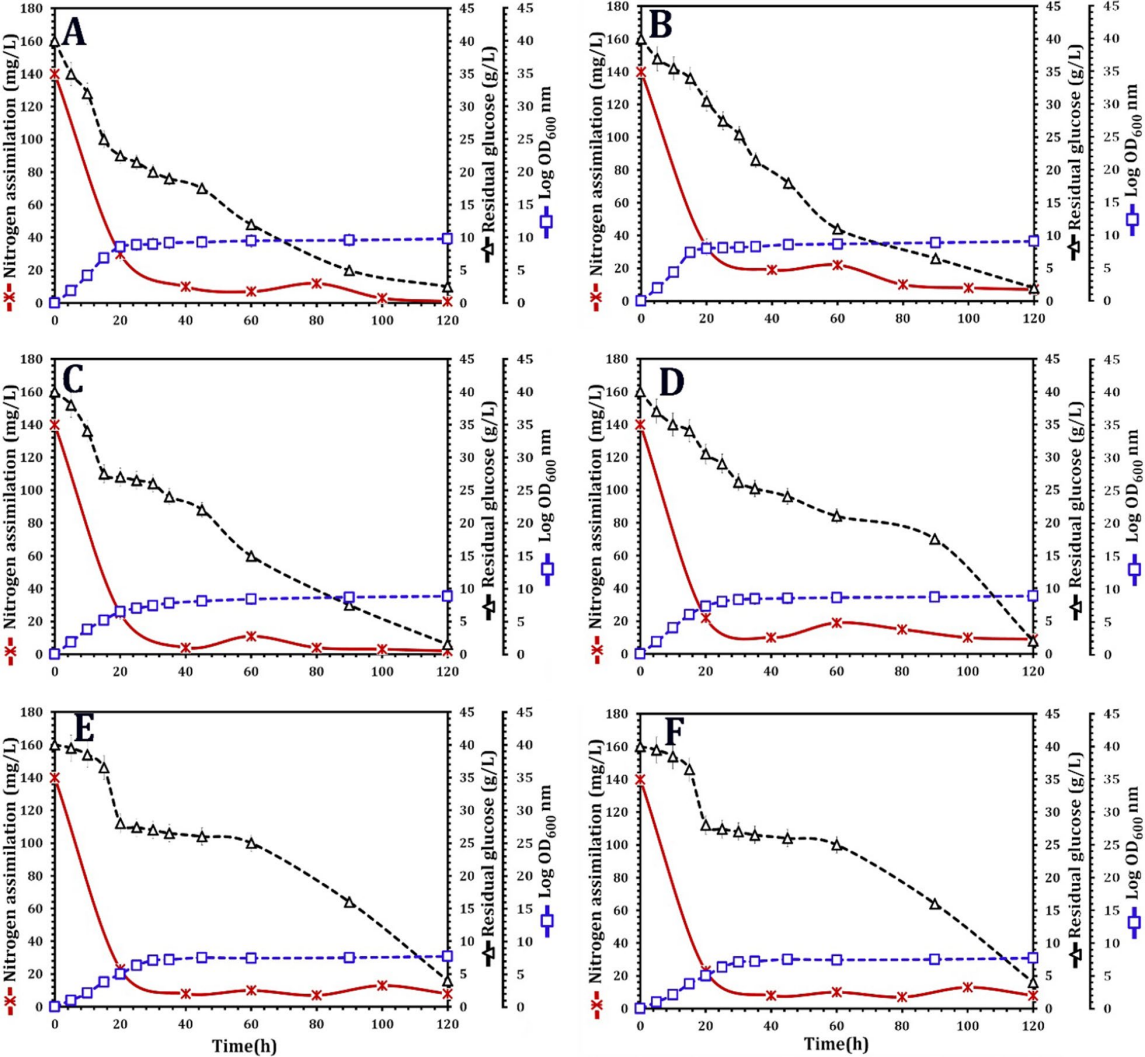
Growth, nitrogen assimilation and residual glucose after 5 days of yeast cultivation in nitrogen-limited glucose-based medium. Four MnP-producing oleaginous yeast strains *Vanrija humicola* strain SSA1514 (**A**), *Meyerozyma caribbica* strain SSA1654 (**B**), *Meyerozyma guilliermondii* strain SSA1547 (**C**), and *Debaryomyces hansenii* strain SSA1502 (**D**), and two MnP-producing non-oleaginous yeast strains *Sterigmatomyces halophilus* strain SSA1655 (**E**), and *Fellozyma inositophila* strain SSA1579 (**F**) are used in this experiment. Values are the mean of three independent replicates, with error bars indicating the standard deviation

Six yeast strains were compared in terms of growth, residual glucose, and nitrogen assimilation (Fig. 3). All oleaginous and non-oleaginous yeast strains were cultured for 5 days at 28 °C under nitrogen limitation in the presence of glucose. All yeast strains displayed typical growth curves, including exponential and stationary phases for the oleaginous yeast strains *V. humicola* SSA1514, *M. caribbica* SSA1654, *M. guilliermondii* SSA1547, and *D. hansenii* SSA1502 (Fig. 3a–d) and the non-oleaginous yeast strains *S. halophilus* SSA1655 and *F. inositophila* SSA1579 (Fig. 3e, f). Nitrogen limitation, as expected, did not greatly boost yeast growth, as evidenced by the evolution of OD_600_. As a result, cells entered the stationary phase after 24 h of growth. Furthermore, as a result of nitrogen limitation, lower glucose uptake rates were found, and hence the profiles of glucose intake followed the drop in nitrogen concentration. Glucose was consumed in that context for up to 120 h of cultivation. After 5 days of incubation at 28 °C, 10–12.7% of glucose remained in *S. halophilus* SSA1655 and *F. inositophila* SSA1579 cultures, but it reduced to 3.9–5.2% in *V. humicola* SSA1514, *M. caribbica* SSA1654, *M. guilliermondii* SSA1547, and *D. hansenii* SSA1502 cultures. After 24 h, however, nitrogen was significantly reduced (86–96.4%) and thereafter remained constant for all oleaginous and non-oleaginous yeasts (Fig. 3).

### Construction of NYC-1 consortium

In this study, a new yeast consortium NYC-1 which stands for the molecularly identified species *M. caribbica* strain SSA1654, *D. hansenii* strain SSA1502, *M. guilliermondii* strain SSA1547, and *V. humicola* strain SSA1514 was successfully developed. The potential of MnP-producing oleaginous yeast consortium members to decolorize textile azo dyes, withstand lignocellulose degradation inhibitors, and produce biodiesel was investigated for multipurpose applications, including bioremediation, lignocellulose valorization, and biofuel production.

### Enzyme activity

**Fig. 4:**
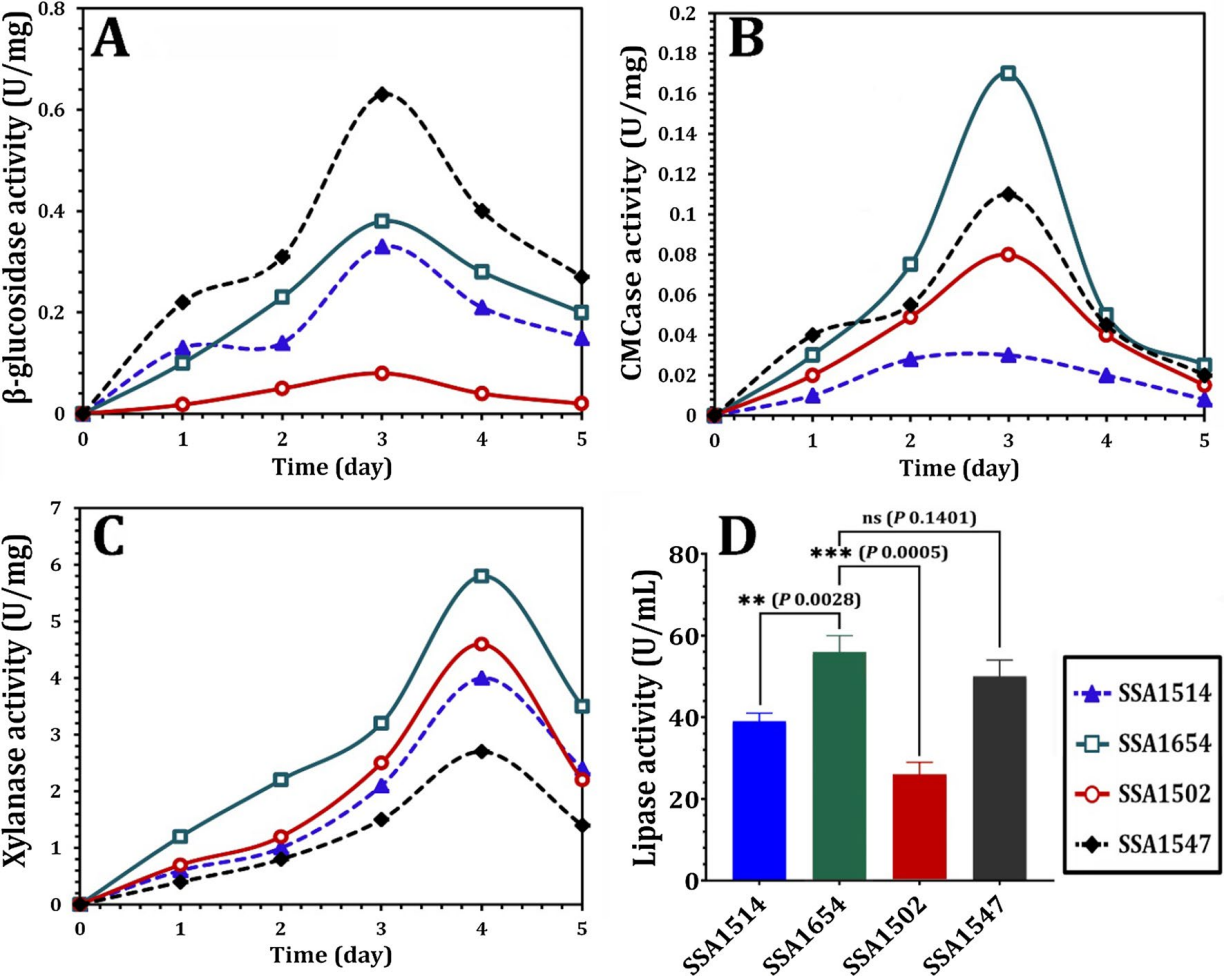
Enzyme activities of the MnP-producing oleaginous yeast strains *Meyerozyma caribbica* strain SSA1654, *Meyerozyma guilliermondii* strain SSA1547, *Debaryomyces hansenii* strain SSA1502, and *Vanrija humicola* strain SSA1514, including β-glucosidase (**A**), CMCase (**B**), xylanase (**C**), and lipase (**D**). Values are the mean of three independent replicates, with error bars indicating the standard deviation. *P* value ≤ 0.05 is significant

The NYC-1 consortium's *M. caribbica* SSA1654, *D. hansenii* SSA1502, *M. guilliermondii* SSA1547, and *V. humicola* SSA1514 strains were examined for their β-glucosidase, CMCase, xylanase, and lipase activities (Fig. 4). Among the lignocellulolytic enzymes tested, *M. guilliermondii* SSA1547 gave a maximum yield of β-glucosidase (0.64 ± 0.07 U/mg) on day 3, whereas *D. hansenii* SSA1502 produced the least β-glucosidase activity (0.061 ± 0.005 U/mg) (Fig. 4a). *M. caribbica* SSA1654 had the highest CMCase yield (0.17 ± 0.03 U/mg) on day 3, while *V. humicola* SSA1514 had the lowest CMCase activity (Fig. 4b). Furthermore, *M. caribbica* SSA1654 had the highest xylanase yield (5.8 ± 0.9 U/mg) on day 4 when compared to *M. guilliermondii* SSA1547, which had the lowest xylanase activity (2.62 ± 0.1 U/mg) on day 4 (Fig. 4c). The time-course analysis, on the other hand, revealed that the maximal lipase synthesis in all yeast strains occurred after 3 days of incubation (data not shown). At the time, the lipase production rates of the yeast strains tested ranged from 25.6 ± 3.2 U/mL for *D. hansenii* SSA1502 to 55.3 ± 5.4 U/mL for *M. caribbica* SSA1654 (Fig. 4d). *M. guilliermondii* SSA1547, *M. caribbica* SSA1654, *D. hansenii* SSA1502, and *V. humicola* SSA1514 produced β-glucosidase, CMCase, xylanase, and lipase activities simultaneously, implying that these MnP- and TAG-producing yeasts could be efficiently employed for the valorization of organic and fatty substrates.

### Tolerance to inhibitors derived from lignocellulose degradation

To investigate the inhibitory effect on growth of the four MnP-producing oleaginous yeast strains comprising the NYC-1 consortium, five representative lignocellulose degradation inhibitors were chosen: furfural, 5-hydroxymethyl furfural (5-HMF), acetic acid, vanillin, and formic acid. As given in Table 3, all yeast strains *M. caribbica* SSA1654, *M. guilliermondii* SSA1547, *D. hansenii* SSA1502, and *V. humicola* SSA1514 could grow in the presence of all inhibitory substances in the tested range. Among the four strains, *M. caribbica* SSA1654 and *M. guilliermondii* SSA1547, showed the highest tolerance to furfural (1.0 g/L), while *M. caribbica* SSA1654 showed the highest tolerance to 5-HMF (2.5 g/L) and vanillin (2.0 g/L). Furthermore, *M. caribbica* SSA1654 could grow in the presence of 2.5 g/L acetic acid but grew moderately. Based on the findings, *M. caribbica* SSA1654 was chosen for future study, because it produced the highest lipid concentration, accumulated the most lipid, and was tolerant to lignocellulosic inhibitory compounds.

**Table 3:** Effect of inhibitors derived from lignocellulose degradation on the growth of the MnP-producing oleaginous yeasts comprising NYC-1 consortium

Compound	Concentration (g/L)	Growth*			
		*M. caribbica* SSA1654	*M. guilliermondii* SSA1547	*D. hansenii* SSA1502	*V. humicola* SSA1514
Furfural	0.5	+ 3	+ 3	+ 3	+ 3
	1.0	+ 2	+ 3	DG	+ 1
	1.5	DG	NG	NG	NG
5-HMF	0.5	+ 3	+ 3	+ 3	+ 3
	1.0	+ 3	+ 3	+ 3	+ 3
	2.0	+ 3	+ 3	+ 3	+ 3
	2.5	+ 3	WG	+ 1	+ 1
Formic acid	0.5	+ 3	+ 3	+ 2	+ 3
	1.0	+ 3	WG	+ 1	DG
	3.0	+ 1	NG	NG	NG
	4.0	NG	NG	NG	NG
Vanillin	0.5	+ 3	+ 3	+ 3	+ 3
	1.0	+ 3	+ 3	+ 3	+ 1
	2.0	+ 1	DG	NG	NG
	2.5	NG	NG	NG	NG
Acetic acid	0.5	+ 3	+ 3	+ 3	+ 3
	1.0	+ 3	+ 3	+ 3	+ 3
	1.5	+ 2	+ 1	+ 1	+ 3
	2.5	+ 1	NG	NG	NG

*NG* no growth, *WG* weak growth, *DG* delayed growth and reached maximum turbidity after 7 days of incubation*,* *+ 3* excellent growth*,* *+ 2* good growth, *+ 1* moderate growth

*Control for all strains tested was + 3, since control is the medium without lignocellulose degradation inhibitor

### Effect of inhibitors derived from lignocellulose degradation on lipid accumulation

The effects of inhibitors derived from lignocellulose degradation, viz. furfural, formic acid, 5-HMF, acetic acid, and vanillin, on the biomass and lipid production of the selected MnP-producing oleaginous yeast strain, *M. caribbica* SSA1654, were investigated in this study (Table 4). The biomass and lipid concentrations in the absence of inhibitory compounds (the control) were 14.65 ± 0.18 and 6.88 ± 1.65 g/L, respectively, representing 47.3% of dry biomass. Clearly, furfural and formic acid had a significant inhibitory effect on biomass production and lipid accumulation by *M. caribbica* SSA1654, compared to the other lignocellulose degradation inhibitors tested, which had a minor effect. Our findings revealed that as formic acid concentrations increased (0.1 to 0.5 g/L), biomass and lipid concentrations decreased gradually, and growth completely stopped when formic acid concentration reached 0.5 g/L. Adding acetic acid at a concentration of 0.1 g/L, on the other hand, improved lipid accumulation (7.11 ± 1.84 g/L) when compared to the control (6.88 ± 1.65 g/L). When acetic acid concentrations were between 0.1 and 0.5 g/L, there was little difference in biomass production, but when acetic acid concentration was 1.0 g/L, there was a significant decrease in biomass production (5.12 ± 0.0 g/L). The lipid accumulation of *M. caribbica* SSA1654 was strongly inhibited by furfural. Its lipid production was clearly reduced even at the lowest tested concentration of furfural (0.05 g/L), with a 55% reduction in lipid production. However, even at concentrations of up to 5.0 g/L, 5-HMF had little effect on biomass production (27%) and lipid accumulation (31%). Similar to 5-HMF, vanillin showed less inhibitory effects on biomass production and lipid accumulation of *M. caribbica* SSA1654 at tested vanillin concentrations (0.1–1.0 g/L).

**Table 4:** Effect of lignocellulose degradation inhibitors on the accumulation of lipid by *M. caribbica* SSA1654

Compound	Concentration (g/L)	Biomass (g/L)	Lipid (g/L)	Lipid content (%, w/w)
Control	0.0	14.65 ± 0.18	6.88 ± 1.65	47.3 ± 1.80
Furfural	0.05	7.35 ± 0.0	2.55 ± 0.0	21.5 ± 1.14
	0.1	5.45 ± 0.13	1.23 ± 0.11	19.4 ± 0.88
	0.2	4.37 ± 0.10	0.52 ± 0.0	10.7 ± 0.91
	0.5	2.17 ± 0.15	0.0 ± 0.0	5.2 ± 0.13
5-HMF	0.1	14.67 ± 0.11	6.80 ± 1.60	45.4 ± 1.13
	0.5	14.35 ± 0.10	6.91 ± 1.17	44.6 ± 1.10
	1.0	14.40 ± 0.12	6.97 ± 1.57	47.9 ± 0.80
	5.0	12.75 ± 0.31	5.46 ± 1.11	48.5 ± 0.31
Formic acid	0.1	14.87 ± 0.11	6.89 ± 1.47	46.34 ± 1.40
	0.2	12.57 ± 0.10	4.39 ± 0.9	42.19 ± 1.82
	0.3	6.64 ± 0.20	0.91 ± 0.3	9.37 ± 0.11
	0.5	0.0 ± 0.0	0.0 ± 0.0	0.0 ± 0.0
Vanillin	0.1	14.55 ± 0.13	6.71 ± 1.55	47.1 ± 1.20
	0.5	12.83 ± 0.21	7.05 ± 1.51	48.4 ± 1.95
	1.0	12.45 ± 0.14	5.87 ± 1.74	47.7 ± 2.21
Acetic acid	0.1	14.75 ± 0.15	7.11 ± 1.84	47.87 ± 2.10
	0.5	14.98 ± 0.31	7.57 ± 1.55	44.21 ± 0.87
	1.0	5.12 ± 0.0	0.92 ± 1.85	10.1 ± 0.12

### Azo dye decolorization performance

**Fig. 5:**
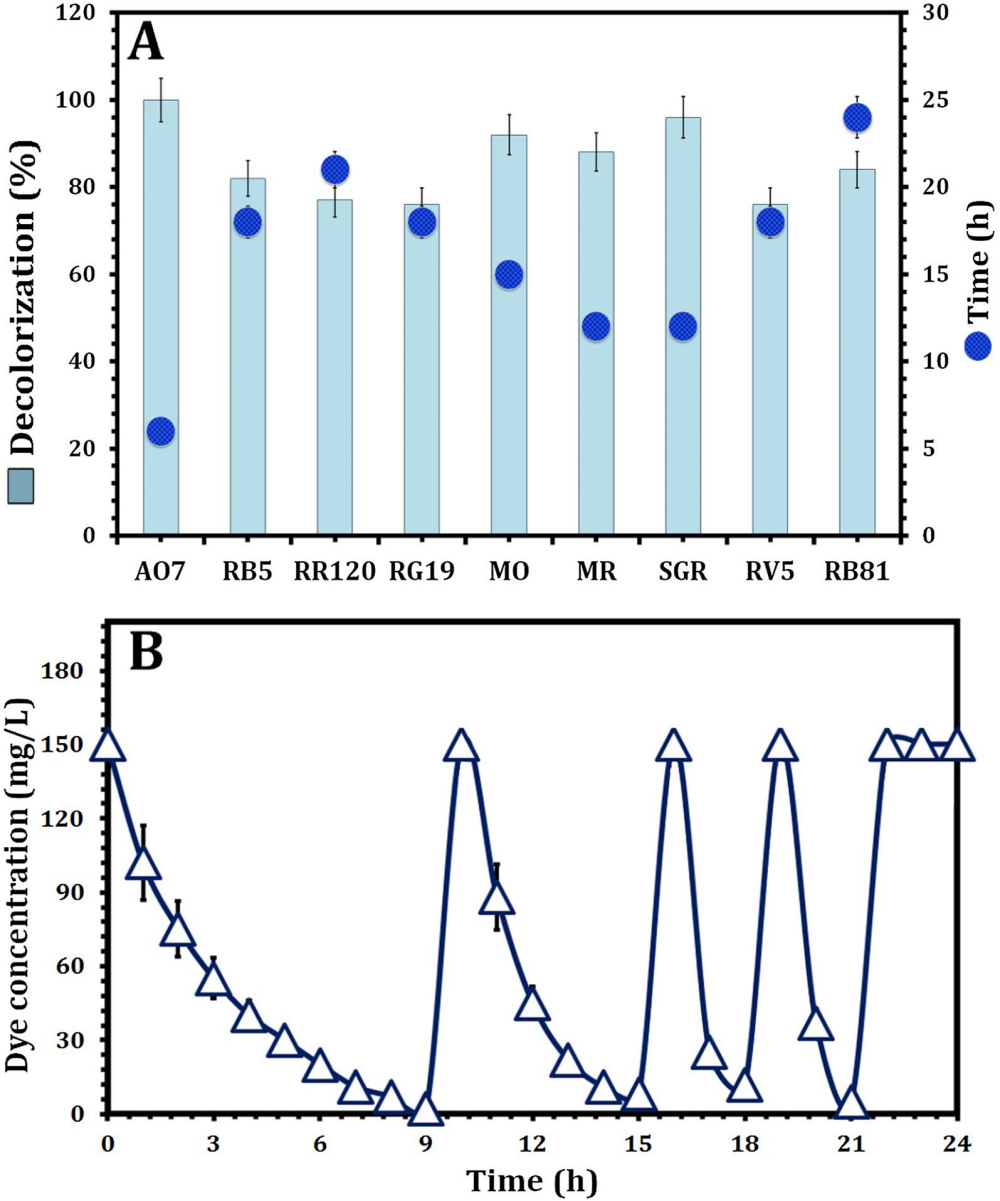
Performance of the constructed MnP-producing oleaginous yeast consortium NYC-1 on decolorizing various azo dyes (**A**) and after repeated addition of 150 mg/L AO7 under static conditions (**B**). *AO7* Acid Orange 7, *RG19* Reactive Green 19, *MO* Methyl Orange, *RB81* Reactive Blue 81, *RR120* Reactive Red 120, *MR* Methyl Red, *RB5* Reactive Black 5, *RV5* Reactive Violet 5, *SGR* Acid Brilliant Scarlet GR

The decolorization of nine azo dyes was studied using the newly constructed MnP-producing oleaginous yeast consortium NYC-1. The constructed NYC-1 consortium, as shown in Fig. 5a, demonstrated effective decolorization of all individual azo dyes tested within 24 h, up to a dye concentration of 250 mg/L, indicating non-specific reduction of the azo bond. However, as the dye concentration increased, the percentage of decolorization decreased significantly, with the percentage of decolorization being 10 times lower at 500 mg AO7/L than at 50 mg/L. The maximum decolorization percentage and time required for dye decolorization clearly differed between the dyes tested (Fig. 5a), probably due to differences in molecular structure. More specifically, azo dyes (AO7; Acid Orange 7, MO; Methyl Orange, RB81; Reactive Blue 81, MR; Methyl Red, and SGR; Acid Brilliant Scarlet GR) with low molecular weight showed a higher decolorization percentage, as compared to azo dyes with complex structure and high molecular weight (RB5; Reactive Black 5, RR120; Reactive Red 120, RG19; Reactive Green 19, and RV5; Reactive Violet 5) (Fig. 5a).

The widespread use of azo dyes in the textile industry results in massive amounts of textile effluents, which are difficult to biodegrade. As a result, it is critical to screen and construct microbial consortia that are potentially suitable for the effective processing of azo dye-loaded industrial effluents. The NYC-1 consortium's efficiency in decolorizing azo dyes in simulated wastewater was evaluated in this study. Clearly, after decolorizing the simulated wastewater for 21 h, the derived peak between 330 and 580 nm completely disappeared (Additional file 1: Fig. S1). As a result, NYC-1 is a promising microbial consortium for effective decolorization of industrial wastewater containing various azo dyes. The efficacy of NYC-1 consortium on azo dye decolorization was compared to other individual and microbial consortia reported earlier (Table 5). The performance of the NYC-1 consortium on decolorization after repeated dye addition, on the other hand, was investigated. As shown in Fig. 5b, the time required to achieve maximum decolorization efficiency after the initial injection of AO7 (150 mg/L) was 9 and 6 h, respectively. At the same time, it had just been 3 h after repeated cycles of dye addition.

**Table 5:** Comparison of MnP-producing oleaginous yeast consortium NCY-1 with other microbial consortia in literature in terms of azo dye decolorization

Consortium	Dye	Concentration (mg/L)	Decolorization (%)	Time (h)	References
Y-BCSH yeast consortium	Reactive Orange 16	100	83	15	Ali et al. [8]
Yeast consortium	Reactive Violet 5	100	71	24	Olteanu et al. [74]
SKB-II bacterial consortium	Congo Red	10	96	150	Tony et al. [75]
Yeast consortium	Reactive Orange 16	100	53	24	Olteanu et al. [74]
Y-BCSH yeast consortium	Reactive Green 19	100	90.6	6	Ali et al. [8]
Bacterial consortium	Reactive Red 120	60	82	24	Khehra et al. [76]
NYC-1 consortium	Acid Orange 7	50	100	6	This study
NYC-1 consortium	Acid Orange 7	100	100	9	This study
NYC-1 consortium	Acid Orange 7	200	100	21	This study
NYC-1 consortium	Acid Orange 7	250	88	24	This study

**Fig. 6:**
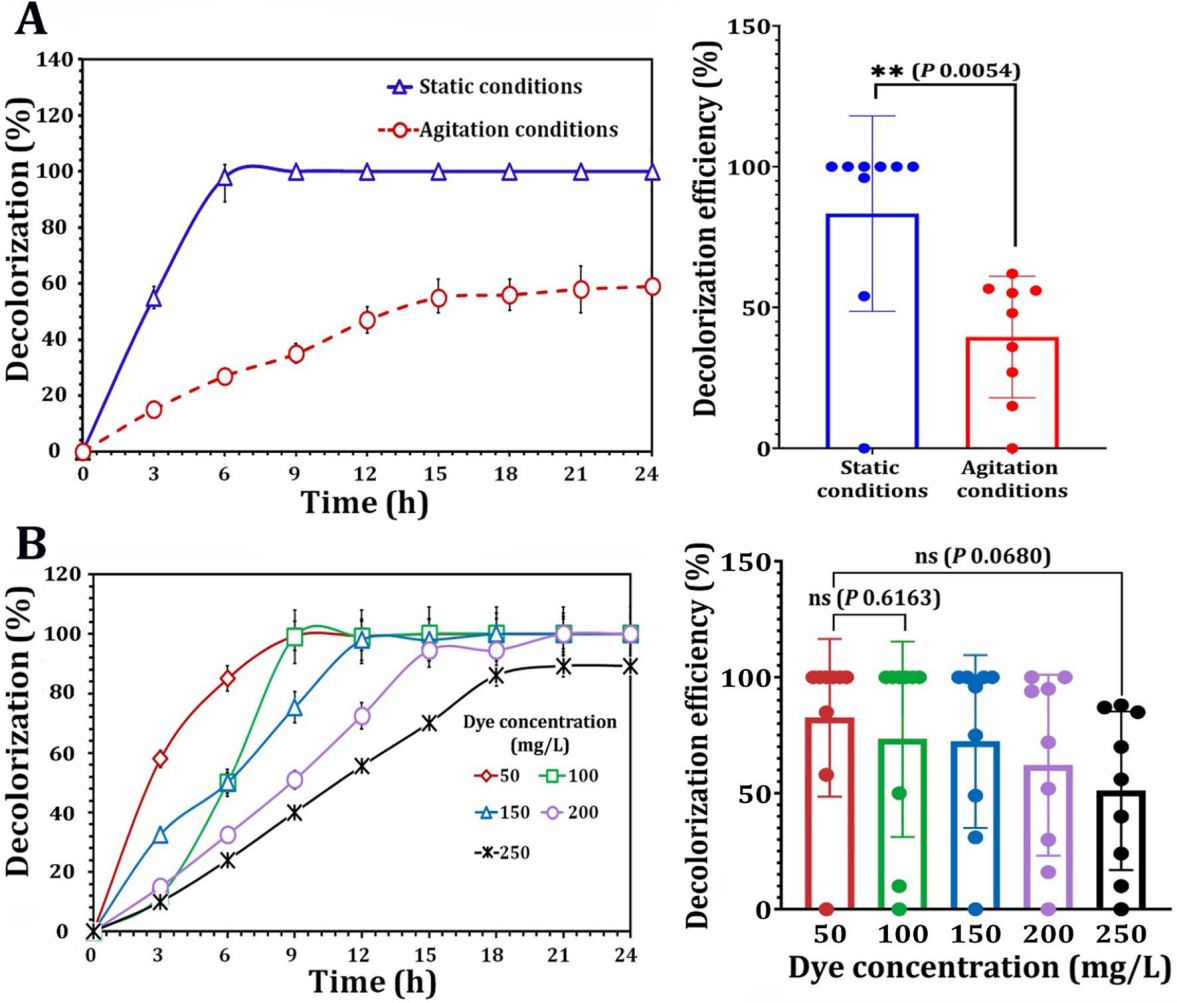
Influence of static and agitation conditions (**A**) and different dye concentrations (**B**) on the decolorization of AO7 by the constructed MnP-producing oleaginous yeast consortium NYC-1. *P* value ≤ 0.05 is significant

To further examine the efficacy of dye biodegradation, the effects of static and agitation conditions on the decolorization performance of AO7 by the MnP-producing oleaginous yeast consortium NYC-1 were investigated. Under static conditions, approximately complete AO7 decolorization was obtained by the NYC-1 consortium after cultivation for 6 h. In comparison, after 15 h of incubation, the decolorization efficiency of AO7 under agitation conditions (120 rpm) reached 49% (Fig. 6a). Furthermore, the effect of initial dye concentration on the AO7 decolorization efficacy  was investigated by the NYC-1 consortium. Within 9 h, the medium supplemented with the model azo dye AO7 (initial 50 mg/L) was decolorized. However, further transfers demonstrated rapid decolorization, with more than 98% of AO7 decolorization acquired within 3 h from the original concentration of 50 mg/L. Even when the initial dye concentration was increased to 250 mg/L, the decolorization efficiency exceeded 92% after 18 h of incubation (Fig. 6b).

**Fig. 7:**
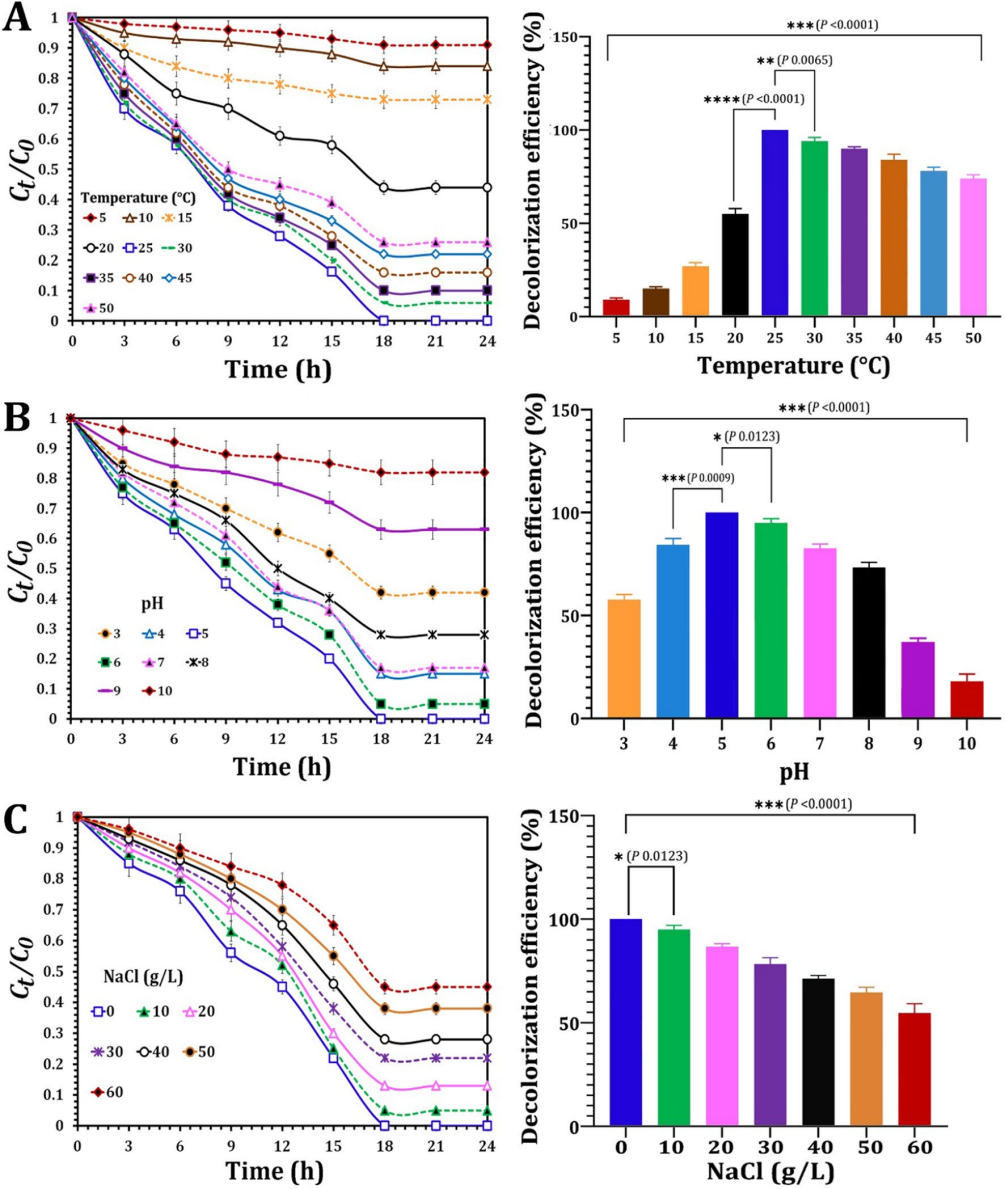
Influence of temperature (**A**), pH (**B**), and salt concentration (**C**) on the decolorization of AO7 by the constructed MnP-producing oleaginous yeast consortium NYC-1. *P* value ≤ 0.05 is significant

The NYC-1 consortium also investigated the influence of several parameters, such as temperature, pH, and salt concentration on the decolorization efficacy of AO7 (Fig. 7). Complete dye decolorization was accomplished after 18 h of incubation at 28 °C, but increasing temperature reduced decolorization efficiency, achieving over 85% decolorization after 24 h of incubation at 50 °C (Fig. 7a). On the contrary, at 5 °C, a significant decrease in decolorization efficiency was determined, with less than 10% decolorization . Thus, the NYC-1 consortium's decolorization effectiveness of AO7 varied significantly with temperature (*p* < 0.0001). Furthermore, as demonstrated in Fig. 7b, the effect of pH change varied. Within 18 h of incubation, the NYC-1 consortium showed complete decolorization of AO7 dye at pH 5. However, as the acidic or alkaline pH increased, the decolorization efficiency declined dramatically, achieving approximately 57 and 15% dye decolorization at pH 3 and 10, respectively. Therefore, the NYC-1 consortium's decolorization effectiveness of AO7 varied considerably with increasing acidity or alkalinity (*p* < 0.0001) (Fig. 7b).

In terms of salt concentration (NaCl up to 60 mg/L), the NYC-1 consortium investigated the decolorization efficiency of AO7 in the presence of high concentrations of NaCl (Fig. 7c). After 18 h of incubation, the dye decolorization efficiency exceeded 95% when the decolorization medium was supplemented with 10 g/L NaCl. However, increasing the salt concentration resulted in a considerable decline in the NYC-1 consortium's AO7 decolorization efficiency, which exceeded 65% when the concentration of NaCl was adjusted at 50 g/L. As a result, the NYC-1 consortium has been identified as a halotolerant consortium that could potentially be applied prior to the disposal of azo dye wastewater, which is also characterized by high salt concentrations (Fig. 7c).

**Fig. 8:**
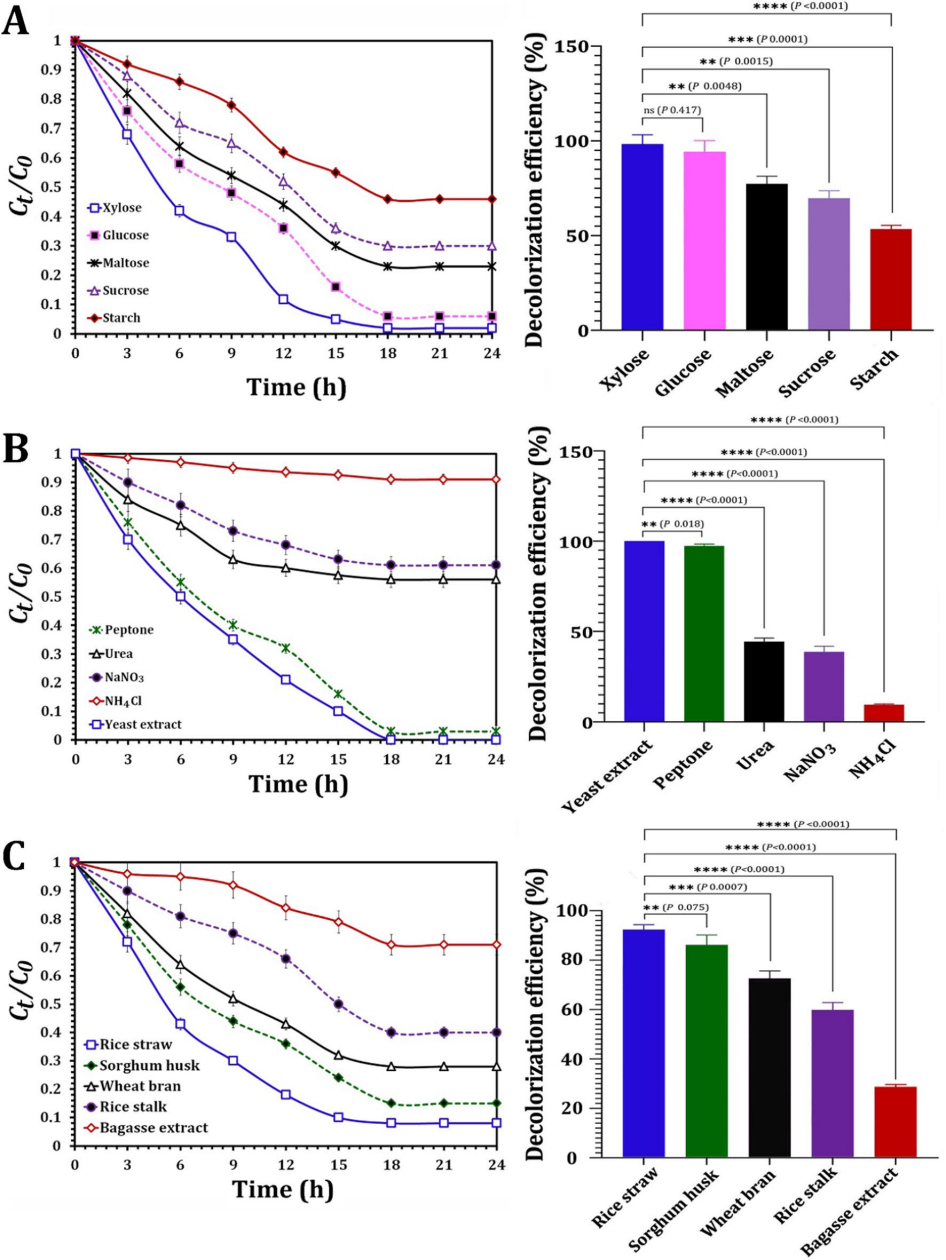
Influence of carbon source (**A**), nitrogen source (**B**), and agricultural wastes (**C**) on the decolorization of AO7 by the constructed MnP-producing oleaginous yeast consortium NYC-1. *P* value ≤ 0.05 is significant

The NYC-1 consortium's decolorization performance against AO7 in the presence of co-substrates (e.g., carbon, nitrogen, or agricultural wastes) was also investigated (Fig. 8). The addition of co-substrates is necessary to improve yeast growth and decolorization of the azo dye. Therefore, the dye decolorization performance of the NYC-1 consortium was investigated in the presence of various carbon (xylose, glucose, maltose, sucrose, starch) and nitrogen (peptone, urea, NaNO_3_, NH_4_Cl, yeast extract) sources at 0.5% (w/v) added to the Bushnell Haas synthetic medium. Furthermore, the effect of various agricultural wastes, such as rice straw and stalk, sorghum husk, wheat bran, and bagasse, on AO7 decolorization was investigated upon adding 0.5 mL extract of 0.5% boiled substrates. In the absence of any supplementation, NYC-1 showed the lowest decolorization activity (approximately 17%) in the synthetic medium. The presence of xylose resulted in the highest percentage of decolorization (98.25%) of the carbon sources tested. In comparison, no significant difference (*p* 0.417) in decolorization efficiency (94.18%) was observed in the presence of glucose. Within 24 h of incubation, the constructed consortium's decolorization efficiency for maltose, sucrose, and starch was 77.34, 69.71, and 53.41%, respectively (Fig. 8a).

In terms of nitrogen sources, yeast extract resulted in complete (100%) decolorization, whereas peptone resulted in 97.2% (Fig. 8b). Urea, NaNO_3_, and NH_4_Cl, on the other hand, inhibited AO7 decolorization by 44.32, 38.80, and 9.67%, respectively, within 24 h. Τhe decolorization efficiency of NYC-1 consortium against AO7 was also evaluated in the presence of agricultural wastes (Fig. 8c). Within 24 h of incubation, rice straw had the highest percentage of decolorization (92.33%), followed by sorghum husk (86.59%). Bagasse extract, on the other hand, showed the lowest decolorization efficiency (28.62%). Rice straw and sorghum husk had a significantly (*p* < 0.0001) greater effect than other agro-wastes used (wheat bran, rice stalk, and bagasse) (Fig. 8c). As a result, using lignocellulosic biomass to improve the decolorization process could be a promising approach while also contributing to the effective management of agro-wastes.

### Influence of azo dye on the fatty acid composition and biodiesel properties

Textile wastewater bioremediation is a new dye treatment technology. Oleaginous yeasts are gaining popularity for their ability to degrade textile wastewater and accumulate fatty acids for biodiesel production. This could be the first study to explore the efficacy of MnP-producing oleaginous yeasts for coupling azo dye decolorization and biodiesel production. The lipids extracted from the AO7-degraded NYC-1 consortium were compared to those extracted from the control experiment (dye-free medium containing NYC-1 yeast consortium). Clearly, there was a decrease in the percentage of saturated fatty acids, particularly dodecanoic acid, as well as an increase in the amount of other alkenes and alkanes in the dye-treated oleaginous yeast consortium compared to the control one (data not shown). The main physicochemical properties of biodiesel produced by the azo dye AO7-degraded NYC-1 consortium were estimated and the results were compared to those obtained from international standards, vegetable oils, and oleaginous yeasts (Table 6). Determining the long-chain saturation factor (LCSF) is essential for estimating critical parameters, such as cetane number, oxidative stability, and kinematic viscosity. Under this scope, the values of LCSF, cetane number, oxidative stability, and kinematic viscosity of biodiesel produced by the AO7-degraded NYC-1 consortium were 4.21, 53, 7.85 h, and 4.38 mm^2^ s^−1^, respectively, while *M. caribbica* SSA1654 achieved biodiesel values of 3.34, 52.32, 8.3 h, and 3.98 mm^2^ s^−1^, respectively (Table 6). Furthermore, when compared to *M. caribbica* SSA1654 (95.85%), *Rhodotorula glutinis* R4 (95.2%), palm oil (64.2%), and olive oil (92.7%), the biodiesel produced by NYC-1 consortium had a degree of unsaturation of 96.12%.

**Table 6:** Estimation of biodiesel properties

Biodiesel properties	International biodiesel standards		Oleaginous yeasts			Vegetable oils
	EN 14214 (Europe)	ASTM D6751-3 (USA)	NYC-1 consortium	*M. caribbica* SSA1654	*R. glutinis* R4	Palm oil
Long chain saturation factor (LCSF)	N.S	N.S	4.21	3.34	2.6	7.7
Cetane number	Minimum 51	Minimum 47	53	52.32	53.0	61.0
Density (g/cm^3^)	0.86–0.9	N.S	N.D	0.88	0.87	N.S
Kinematic viscosity (mm^2^/s)	3.5–5.0	1.9–6.0	4.38	3.98	3.85	4.5
Iodine value (gI_2_/100 g)	Maximum 120	N.s	95.18	92.37	90.0	57.0
Oxidative stability (h)	Minimum 3	Minimum 6	7.85	8.3	10.02	4.0
Saponification value (mg KOH/g-oil)	N.S	N.S	211.4	199.4	203.3	N.D
Degree of unsaturation (%, wt)	N.S	N.S	96.12	95.85	95.2	64.2
Linolenic acid (C18:3)	≤ 12	N.S	4.11	4.07	4.23	0.6

*N.S.* not specified, *N.D.* not determined

Data of *Rhodotorula glutinis* R4 and Palm oil obtained from Maza et al. [93] and Ramos et al. [94], respectively

The fatty acid profiles of four oleaginous yeast strains were determined: *V. humicola* SSA1514, *M. caribbica* SSA1654, *M. guilliermondii* SSA1547, and *D. hansenii* SSA1502, with only the profile of *M. caribbica* SSA1654 presented in this study (Additional file 1: Table S1). *Meyerozyma caribbica* SSA1654 had a high lipid content of 47.25 ± 1.84% (w/w) and a glucose and nitrogen assimilation rates of 40 g/L and 137.5 mg/L, respectively, resulting in an imbalanced metabolism and glucose conversion into neutral lipids. The profile of the extracted lipid of *M. caribbica* SSA1654 in terms of the main lipid classes, monoacylglycerol (MAG), diacylglycerol (DAG), TAG, and free fatty acids (FFA), was assessed qualitatively using thin layer chromatography (TLC). The results were compared with those obtained from the vegetable oil (palm oil) (Additional file 1: Table S1). TLC analysis revealed that the lipid bodies of *M. caribbica* SSA1654 are mostly TAG. Interestingly, the oleaginous yeast flow rate and composition were comparable to those of palm oil. The fatty acid profile of the oleaginous yeast *M. caribbica* SSA1654 was determined after it was grown at a C/N ratio of about 40. Long-chain fatty acids were abundant in the lipid generated under these conditions, particularly oleic acid (C18:1; 60.73% w/w) and palmitic acid (C16:0; 17.34% w/w). The prominent FAMEs were C18, which included stearic acid (C18:0), oleic acid (C18:1), linoleic acid (C18:2), and linolenic acid (C18:3), accounting for 76.22 percent of total FAMEs. On the other hand, total C16, including palmitic acid (C16:0) and palmitoleic acid (C16:1), accounted for 18.94% of total FAMEs (Additional file 1: Table S1). *M. caribbica* SSA1654 also synthesized more mono-unsaturated fatty acid (MUFA; MUFA; 62.33%) than saturated (SFA) and poly-unsaturated fatty acid (PUFA), 19.2% and 13.63%, respectively (Additional file 1: Table S1). Overall, the FAMEs profile, as well as the relative abundance of UFA and SFA, greatly influence biodiesel quality. As a result, *M. caribbica* SSA1654 is a good candidate for third-generation biodiesel production. The high MUFA is advantageous, because it improves the cold filter plugging point. High SFA, on the other hand, results in a high oxidative stability value. As a result, controlling the physicochemical properties of biodiesel with the optimal ratio of SFA to MUFA in FAMEs is critical. Furthermore, the produced biodiesel by *M. caribbica* SSA1654 and NYC-1 consortium had a C18:3 content (less than 12), which was in accordance with the international biodiesel standard EN 14214 (Table 6). Therefore, the MnP-producing oleaginous yeast consortium NYC-1 and the individual Mnp-producing oleaginous yeast strain *M. caribbica* SSA1654 could be novel biological candidates with promising biodiesel production potential.

## Discussion

The decolorization of textile dye wastewater and subsequent lipid production has been highly demanded by screening MnP-producing yeasts from WFTs. Within 120 h, nine of 38 yeast isolates demonstrated the highest peroxidase potential, up to 27 U/mL. Recently, MnP was patented for enzymatic hydrolysis of lignocellulosic substances [48], with ligninolytic enzymes, such as MnP, LiP, Lac, and dye-decolorizing peroxidase, which are commonly derived from various white-rot basidiomycetes and effectively used in the degradation of lignin and other xenobiotics [49]. MnP-producing yeasts have been shown to be highly effective at converting textile azo dye wastewater into innocuous end products [50]. Ligninolytic yeasts produce a variety of peroxidases, which are used in lignin degradation, dye decolorization, and phenolics removal [51]. Biomass digestion by termites, on the other hand, is far more efficient, typically achieving over 95% within a day [37, 52]. *Sterigmatomyces halophilus* strain SSA-1575, *S. halophilus* strain SSA1511 [35], *Barnettozyma californica* strain SSA1518 [35], *Yarrowia* sp. strain SSA1642 [35], and *Meyerozyma guilliermondii* strain SSA1522 [36] have all been tested for azo dye decolorization. However, in the current study, the efficacy of MnP-producing oleaginous yeasts isolated from WFTs for coupling azo dye decolorization and biodiesel production was first explored.

Nile red, a common hydrophobic red fluorescent dye with metachromatic properties, was applied to detect intracellular neutral lipids. In this context, seven MnP-producing yeast strains: *C. stauntonica* SSA1653, *M. caribbica* SSA1654, *D. hansenii* SSA1502, *V. humicola* SSA1514, *M. guilliermondii* SSA1547, *S. halophilus* SSA1655, and *F. inositophila* SSA1579, showed high yellow-gold coloration using Nile red staining. Between them, *M. guilliermondii* strain B1281A and *M. caribbica* strain DMKURK258 can be considered potential biodiesel production sources [53]. Similarly, the oleaginous yeast *D. hansenii* isolated from kefir showed a high ability to accumulate neutral lipids [54], while *C. humicola* strain UCDFST 10–1004 has been previously reported to convert lignocellulose into neutral lipids, easily trans-esterified to biodiesel [55].

Out of the 1600 yeast species known, approximately 100 are oleaginous, with lipid content greater than 20% (w/w) in the form of neutral lipids, primarily TAG [56]. Oleaginous yeasts are promising micro-organisms that can use a variety of carbon sources to synthesize lipids, which can then be used to produce biodiesel. In this study, the accumulation of TAG in the oleaginous yeast strains varied between 33.9 and 47.2%, since *D. hansenii* SSA1502 (33.96 ± 5.72%, w/w), *V. humicola* SSA1514 (43.46 ± 5.34%, w/w), *M. guilliermondii* SSA1547 (44.74 ± 6.09%, w/w), and *M. caribbica* SSA1654 (47.25 ± 1.84%, w/w) showed high lipid-producing ability. Compared to the oleaginous yeasts, the highest concentration of biomass (9.27 ± 0.25 to 11.23 ± 0.71 g/L), lipid production (0.96 ± 0.09 to 1.82 ± 0.61 g/L), lipid content (10.35 ± 1.07 to 10.41 ± 1.14%, w/w), Y_L/x_ (0.104 ± 0.009 to 0.173 ± 0.02 g/g), Y_x/s_ (0.28 ± 0.05 to 0.39 ± 0.01 g/g), Y_L/s_ (0.05 ± 0.01 to 0.09 ± 0.01 g/g), Q_x_ (0.08 ± 0.001 to 0.12 ± 0.005 g/L/h) and Q_L_ (0.005 ± 0.001 to 0.02 ± 0.006 g/L/h) were achieved by the non-oleaginous yeast strains *F. inositophila* SSA1579, *C. stauntonica* SSA1653, and *S. halophilus* SSA1655, while the oleaginous yeasts *Cutaneotrichosporon curvatum* and *Cyberlindnera saturnus* showed the highest lipid content of 36.9 and 33.9% (w/w), respectively [57]. The lipid production of MnP-producing oleaginous yeast strains was compared to that of other oleaginous yeasts reported in the literature [7, 58–61].

In terms of nitrogen consumption, almost complete removal was observed within 24 h of growth, with residual glucose of approximately 15% and assimilation reaching up to 96%. Nitrogen limitation via the de novo synthesis pathway is thought to be the most effective [62]. AMP-deaminase converts adenine-monophosphate (AMP) to inosine-monophosphate (IMP) during nitrogen deficiency. As a result, the production of α-ketoglutarate from isocitrate is impossible, and the citrate that enters the cytoplasm produces acetyl-Co-A. Finally, the fatty acid chains C14–C16 are formed [63]. In contrast to oleaginous yeasts, conventional or non-oleaginous yeasts convert excess carbon into mannan and glucan when nitrogen is limited [64].

The CMCase and xylanase activities produced by *M. caribbica* SSA1654 were maximum at 0.17 ± 0.03 and 5.8 ± 0.9 U/mg on days 3 and 4, respectively, while the β-glucosidase activity produced by *M. guilliermondii* SSA1547 was maximum (0.061 ± 0.005 U/mg) on day 3. The lipase activity of *D. hansenii* SSA1502 ranged from 25.6 ± 3.2 U/mL to 55.3 ± 5.4 U/mL for *M. caribbica* SSA1654. Other oleaginous yeasts with similar CMCase, β-glucosidase, xylanase, and lipase activities have previously been reported, including *Cryptococcus curvatus* [65], *Trichosporon mycotoxinivorans* [66], *Sterigmatomyces halophilus* [35], *Barnettozyma californica* [34], and *Yarrowia* sp. [36]. As a result, the simultaneous production of CMCase, β-glucosidase, xylanase, and lipase by the MnP-oleaginous yeast strains tested in this study may serve as a biocatalyst for biocatalyzing the valorizing of agro-industrial biomass/fatty wastes and subsequent biofuel production.

Not only fermentable sugars, but also a variety of byproducts are produced and discharged into the hydrolysates during the pretreatment and hydrolysis of lignocellulosic biomass. These compounds may have a deleterious impact on micro-organism growth, metabolism, and product production [67]. The existence of inhibitory chemicals in lignocellulosic hydrolysates may be divided into three major categories depending on their origin: weak acids, phenolic compounds, and furan derivatives [68]. However, the concentrations of these chemicals vary depending on the feedstock as well as the pretreatment and hydrolysis processes [55]. As a result, five representative lignocellulose degradation compounds were chosen to investigate their inhibitory effects on growth of the four MnP-producing oleaginous yeast strains comprising the NYC-1 consortium (*M. caribbica* SSA1654, *M. guilliermondii* SSA1547, *D. hansenii* SSA1502, and *V. humicola* SSA1514): furfural from dehydration of pentose sugars; 5-HMF from dehydration of hexose sugars; vanillin from lignin breakdown during acid hydrolysis; acetic acid from de-acetylation of hemicellulose; and formic acid from furfural and 5-HMF breakdown [68]. Among the four strains, *M. caribbica* SSA1654, showed the highest tolerance to furfural (1.0 g/L), 5-HMF (2.5 g/L), vanillin (2.0 g/L) and acetic acid (2.5 g/L). These findings demonstrated that tolerance to lignocellulose degradation inhibitors was affected by the nature and concentration of the compounds, as well as the micro-organisms [69].

Extensive research has been conducted on the effects of lignocellulose degradation inhibitors on ethanol fermentation, lipid accumulation, and oleaginous yeast growth, with the conclusion that lignocellulose degradation inhibitors had a greater impact on yeast growth than lipid accumulation [69]. The uncoupling and intracellular anion accumulation hypotheses explain the inhibitory effects of weak acids. Strong acids are lipid-soluble and can diffuse across the plasma membrane, dissociating due to neutral cytosolic pH, lowering intracellular pH and reducing ATP levels (uncoupling theory) or inhibiting enzyme activity (intracellular anion accumulation theory) [70]. Due to its higher acidity (pKa 3.75) and smaller molecular size (pKa 4.75), formic acid has a stronger inhibitory effect than acetic acid [70]. Furfural and 5-HMF are major degradation products from xylose and glucose, respectively. They are major inhibitors of ethanol fermentation [71]. These compounds inhibit the glycolytic enzymes hexokinase and glyceraldehyde-3-phosphate dehydrogenase [70]. In this study, furfural had strong inhibitory effect on lipid accumulation of *M. caribbica* SSA1654. Enzyme matrices and selective barriers are affected by phenolic compounds, such as vanillin [70]. According to Chandel et al. [72], higher molecular weight phenolic compounds are less harmful to micro-organisms. Our findings are in accordance with Zhao et al. [73] who found that lignocellulose degradation compounds are toxic in high concentrations.

In terms of dye decolorization, azo dyes with low molecular weight demonstrated a higher maximum decolorization percentage by the newly constructed MnP-producing oleaginous yeast consortium NYC-1 than azo dyes with complex structure and high molecular weight. The performance of NYC-1 consortium was compared to that of other consortia reported in the literature [8, 74–76]. It has been reported that simple structured dyes of low molecular weight show increased decolorization rates than complex structures and dyes of high molecular weight [77]. Furthermore, the number of azo bonds, Van der Waals forces, hydrogen bonds, and hydrophobic/electrostatic interactions all have an effect on the rate of dye decolorization [78]. However, after the fourth addition, the constructed consortium's AO7 decolorization performance was comparable. When nutrients are scarce, exponential yeast growth ceases, and microbial death occurs gradually. As a result, the yeast cell's enzyme system is gradually inhibited [79].

Under static conditions, AO7 decolorization by the NYC-1 consortium was significantly higher (*p* 0.0047) than under agitation conditions. The findings support the NYC-1 consortium's preference for static conditions for AO7 decolorization. Azoreductase, the enzyme that catalyzes azo dye decolorization, is inhibited by aeration due to competition between the azo group and oxygen (as an electron acceptor) in the oxidation of reduced electron carriers, such as NADH and quinones [80]. The azo bond in azo dyes is known to be electron-withdrawing. As a result, it reduces the susceptibility of dye molecules to oxidation [78]. In terms of increasing initial dye concentrations, similar results were obtained in the NYC-1 consortium's decolorization efficiency against AO7. At a dye concentration of 250 mg/L, the decolorization efficiency exceeded 92% in 18 h. Furthermore, there was no difference in the pH of the culture medium before and after AO7 decolorization, indicating that dye decolorization can be attributed to the biotic performance of the NYC-1 consortium rather than pH variation in the culture medium [34]. Overall, the presence of azo dyes in aquatic bodies and effluents, ranging from 10 to 200 mg/L, is hazardous and aesthetically unappealing [81, 82]; thus, azo dye degradation is critical prior to discharge.

The effects of temperature, pH, and salt concentration on dye decolorization performance were also investigated by the NYC-1 consortium. The developed consortium demonstrated significantly higher decolorization efficiency at 28 °C than at 20 °C (*p* < 0.0001) and 30 °C (*p* 0.0065). In addition, at 5 °C, the decolorization efficiency dropped sharply to less than 10%, confirming previous findings of dye decolorization inhibition at low temperatures [83]. Furthermore, the NYC-1 consortium retained its decolorizing efficiency at high temperatures (up to 50 °C) in the current study, demonstrating its valuable potential for azo dye bioremediation. The NYC-1 consortium also achieved complete AO7 decolorization at pH 5 within 18 h of incubation, whereas decolorization efficiency decreased significantly with increasing acidity (pH 3; 57% decolorization) or alkalinity (pH 10; 15% decolorization). The NYC-1 consortium demonstrated significantly higher decolorization efficiency at pH 5 than at pH 4 (*p* 0.0009) and pH 6 (*p* 0.0123). The low dye decolorization efficiency at higher acidic or alkaline pH values was most likely due to a change in the dye chemical structure caused by the formation of protonated azo dyes [84]. Furthermore, after 18 h of incubation, the dye decolorization efficiency exceeded 95% in the presence of 10 g/L NaCl. However, increasing the salt concentration resulted in a significant decrease in decolorization efficiency (*p* < 0.0001), with 50% decolorization achieved at 60 g/L NaCl. As a result, the NYC-1 consortium represents a highly promising halo-tolerant yeast consortium with significant potential for bioremediation of textile wastewater containing azo dyes and high salt concentrations [85].

It has been reported that the addition of co-substrates (e.g., carbon, nitrogen, or agricultural wastes) is required for both yeast growth and improving azo dye decolorization performance [86]. Decolorization performance took its highest value in the case of xylose (98.25%) with a non-significant difference with glucose (94.18%; *p* 0.417). However, both xylose and glucose revealed significant differences between maltose (*p* 0.0048) and sucrose (*p* 0.0015). In addition, xylose, glucose, maltose, and sucrose showed a significantly higher decolorization performance (*p* 0.0001) when compared with starch. Although the isolated bacteria's conversion of glucose to organic acids may inhibit textile dye decolorization [87], the presence of glucose or xylose can aid AO7 dye decolorization by the NYC-1 consortium. Due to the microbial preference for these added carbon sources, Saratale et al. [88] found that adding di- and polysaccharides is less effective in promoting decolorization efficiency than monosaccharides. On the other hand, organic and inorganic nitrogen sources are essential nutrients for yeast growth [86]. The NYC-1 consortium determined that yeast extract (100%) and peptone (97.2%) are the best nitrogen sources for AO7 decolorization, because both yeast extract and peptone provide significantly higher decolorization efficiency (*p* < 0.0001) than other nitrogen sources tested (urea, NaNO_3_, and NH_4_Cl). It has been reported that peptone and yeast extract are responsible for activating NADH expression and thus effectively decolorizing the dye [88]. The low decolorization efficiency in the presence of NH_4_Cl can be attributed to its inhibitory effect on the performance of the decolorization enzymes [89]. Finally, the NYC-1 consortium investigated the decolorization efficiency of AO7 in the presence of agro-waste extracts. Rice straw demonstrated the highest decolorization performance (92.33%) compared to bagasse extract (28.62%), owing to the production of volatile organic acids or alcohols that could serve as electron donors for dye reduction. As a result, the addition of agro-wastes to improve AO7 decomposition is an environmentally friendly and low-cost process that addresses the issue of vast quantities of agro-wastes produced globally.

TLC is a tool for monitoring the conversion of extracted lipids to FAMEs during the transesterification reaction in the production of biodiesel [90]. According to the TLC results obtained in this study, both *M. caribbica* SSA1654 and palm oil standard had a similar flow rate and composition. Furthermore, three types of neutral lipids, MAG, DAG, and TAG, have been identified in oleaginous yeasts, with TAG remaining the most abundant [91]. Therefore, the TAG produced by *M. caribbica* SSA1654 could be valuable when aiming at biodiesel production. In contrast, Patel et al. [92] reported that lipid-producing yeasts in the presence of glucose and under nitrogen limitation mainly synthesize myristic acid (C14:0), palmitic acid (C16:0), stearic acid (C18:0), oleic acid (C18:1), linoleic acid (C18:2), and linolenic acid (C18:3). Furthermore, the total SFA, MUFA, and PUFA exceed 95% of the total FAMEs, similar to vegetable oils mainly comprising of C16 and C18 fatty acids (SFA or MUFA) [43, 93, 94], suggesting that *M. caribbica* SSA1654 could be a potential oleaginous yeast, isolated from WFTs, valued for biodiesel production.

The physicochemical properties of biodiesel derived from NYC-1 consortium and *M. caribbica* SSA1654 lipids were compared to those obtained from oleaginous yeasts [93] and vegetable oils [94]. Increased LCSF has been shown to have a negative effect on the cold filter plugging point (cold flow behavior) of biodiesel [95]. The crystallization of biodiesel in the engine pipeline under cold conditions is a significant challenge [94]. The degree of unsaturation of fatty acids determines oxidative stability, which is another critical criterion used to determine the shelf-life of any fuel [95]. The cetane number of the biodiesel produced by *M. caribbica* SSA1654 and the NYC-1 consortium met the EN 14,214 (minimum 51) and ASTM 6751–3 values (minimum 47). According to Hoekman et al. [96], there are two limit values for cetane numbers. The higher value reduces engine efficiency, while low cetane number values result in high hydrocarbon emissions.

## Conclusion

Lignocellulose biomass and textile azo dyes are recalcitrant substrates that pose a challenge for microbial decomposition. Several microorganisms, on the other hand, exhibit remarkable enzymatic activity and have a high potential for wastewater treatment and simultaneous bioenergy production. *V. humicola* SSA1514, *M. caribbica* SSA1654, *M. guilliermondii* SSA1547, and *D. hansenii* SSA1502 were the most advantageous lipid-accumulating strains, exhibiting MnP, cellulase, xylanase, and lipase activities, as well as tolerance to common lignocellulose degradation inhibitors. Among the four strains, *M. caribbica* SSA1654, showed the highest tolerance to furfural, 5-HMF, vanillin and acetic acid. Furfural and formic acid had a significant inhibitory effect on biomass production and lipid accumulation by *M. caribbica* SSA1654, compared to the other lignocellulose degradation inhibitors tested, which had a minor effect. The decolorization of AO7 azo dye by the newly constructed MnP-producing oleaginous yeast consortium NYC-1 was investigated, and the results showed nearly complete decolorization, particularly in the presence of xylose and yeast extract, which were used as carbon and nitrogen sources, respectively. Simultaneously, rice straw supplementation resulted in 92.3% AO7 decolorization. The produced biodiesel by *M. caribbica* SSA1654 and NYC-1 consortium had a C18:3 content, which was in accordance with the international biodiesel standards. Overall, the enzymatic performance of yeasts and their high lipid content indicate that they have great potential for the effective valorization of lignocellulosic waste and industrial wastewater containing azo dyes, as well as the production of biodiesel. However, more research is required before a large-scale and environmentally friendly application can be established.

## Methods

### Dyestuff and agricultural wastes

**Fig. 9:**
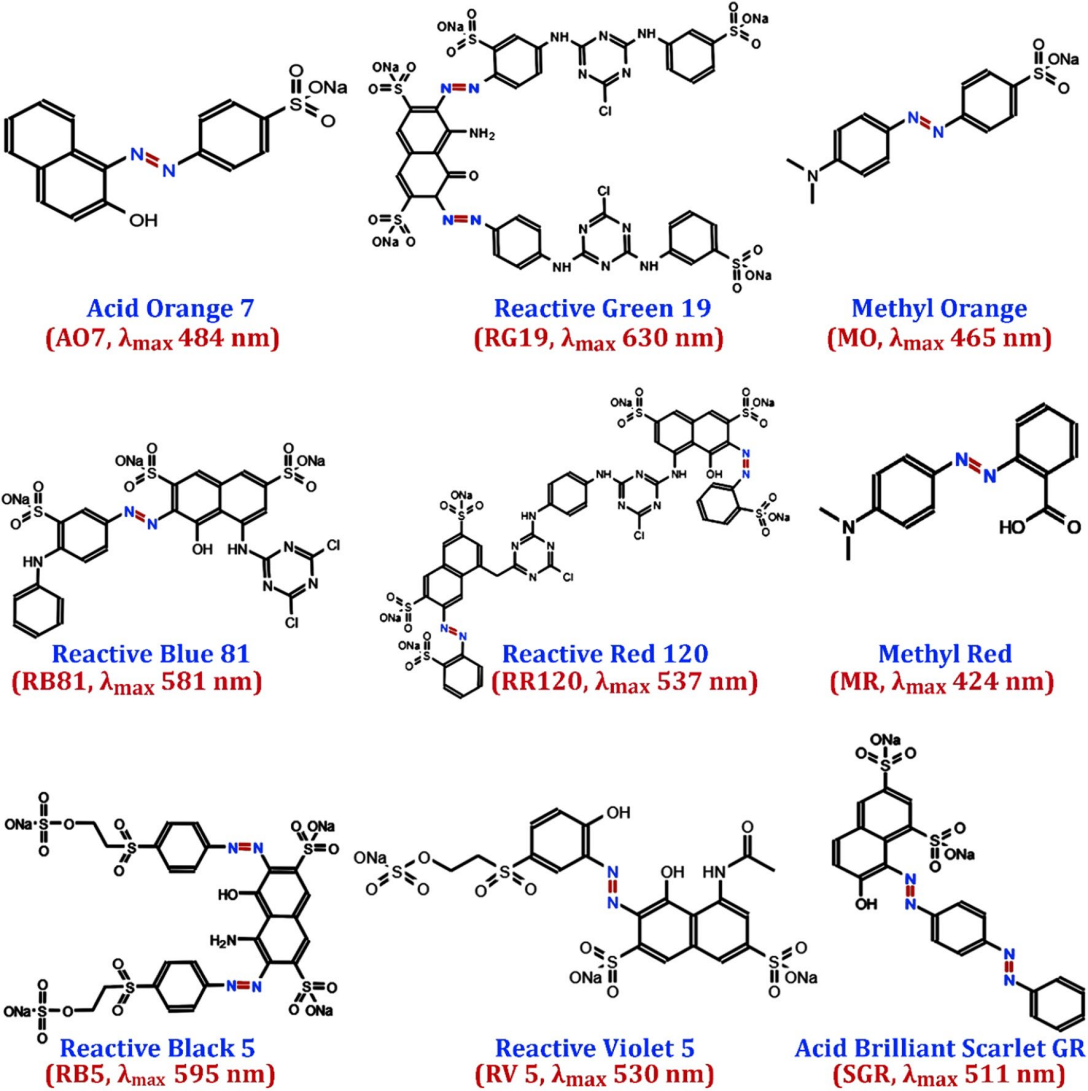
Chemical structure of various azo dyes used in this study and their corresponding maximum wavelengths (λ_max_)

Nine azo dyes (Sigma-Aldrich, St. Louis, USA) were used in this study. The names of these dyes are Reactive Green 19, Methyl Red, Methyl Orange, Reactive Blue 81, Reactive Red 120, Reactive Violet 5, Acid Orange 7, Acid Brilliant Scarlet GR, and Reactive Black 5. The chemical structure of dyes and their corresponding maximum wavelengths (λ_max_) are given in Fig. 9. In this work, several agricultural wastes (rice straw, bagasse, wheat bran, rice stalk, and sorghum husk) were obtained from local farmers and industries (Zhenjiang, China) to evaluate their impacts on the AO7 dye decolorization by the developed yeast consortium. The agricultural waste extracts were prepared in the Bushnell Haas medium (5.0 mL extract of 0.5% boiled agricultural residue).

### Isolation and screening of oleaginous yeasts capable of azo dye decolorization

**Fig. 10:**
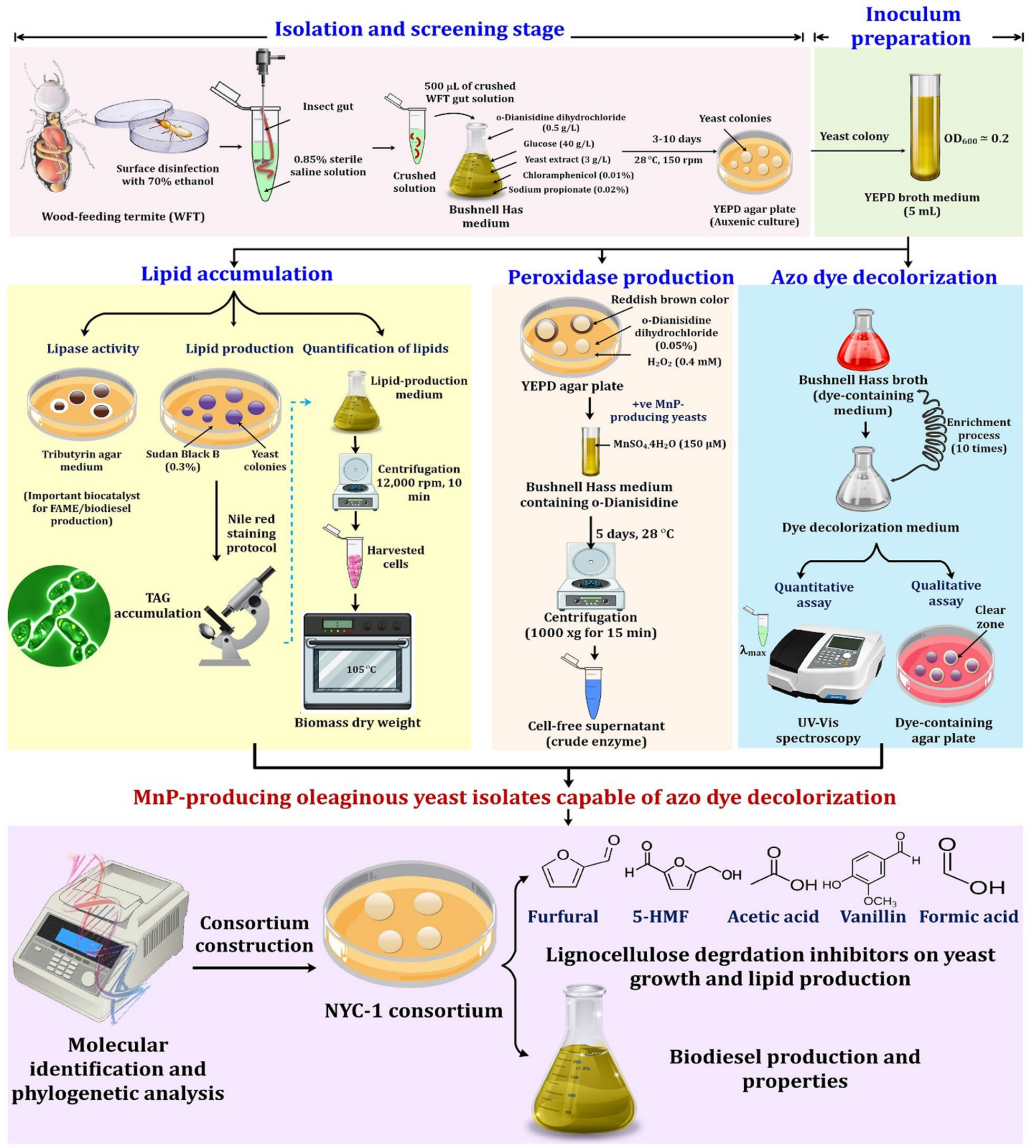
Experimental set up for isolation and screening MnP- producing yeasts inhabiting wood-feeding termite gut symbionts intended for azo dye decolorization, tolerance to lignocellulose degradation inhibitors, and biodiesel production

The experimental setup for the isolation and screening of MnP-producing oleaginous yeasts intended for azo dye decolorization and biodiesel production is depicted in Fig. 10. The WFTs, *R. chinenesis* and *C. formosanus* were collected from rotting wood trees at Wuhan and Nanjing, China as reported previously [32, 47]. Isolation of yeasts from the gut symbionts of *R. chinenesis* and *C. formosanus* was performed following Suh and Blackwell [97]. The surface of insect samples was disinfected twice with 70% ethanol for 1 min prior to dissection. The insect guts were then eliminated aseptically and homogenized in a sterile saline solution (0.85% NaCl, w/v) [23]. Isolation experiments were conducted in conical flasks (100 mL) with a working volume of 40 mL of the Bushnell Hass medium using aliquots of 500 µL crushed WFT gut solutions [24]. This synthetic medium contained (g/L): 2.45 NaH_2_PO_4_, 6.8 KH_2_PO_4_, 1.72 MgSO_4_.7H_2_O, 0.067 MnSO_4_.7H_2_O, and 0.2 CaCl_2_.2H_2_O. The medium was also supplemented with 0.5 g/L o-Dianisidine dihydrochloride (as a precursor of many azo dyes as well as a peroxidase substrate) and glucose at a final concentration of 40 g/L. Yeast extract (3.0 g/L) was included as the sole nitrogen source and yielding a C/N ratio of around 40. As antibacterial and antifungal agents, 0.01% chloramphenicol and 0.02% sodium propionate were added to the medium. The flasks were then incubated for 3–10 days at 28 °C under shaking conditions of 150 rpm. Repeated sub-culturing on yeast extract–peptone–dextrose (YEPD) medium was used to isolate distinct yeast colonies with different morphotypes. The YEPD medium contained (g/L): 20 peptone, 10 dextrose, 10 yeast extract, and 20 agar. Inoculum preparation of purified yeast isolates was prepared as shown in Fig. 10.

### Lipid accumulation

The ability of yeast isolates to accumulate TAG was qualitatively assessed using Sudan Black B staining protocol [98] by incubating the isolates for 30 min, and observing blue color retention after rinsing with 70% ethanol. For TAG (lipid) accumulation among oleaginous and non-oleaginous yeasts, the selected isolates were examined under a fluorescence microscope (Olympus BX35) at 460–500 nm following the Nile red staining protocol [46]. The most promising oleaginous yeasts found to be positive for Sudan Black B were tested for lipase activity on the agar plates of tributyrin medium (10 g/L tributyrin, 3 g/L yeast extract, 5 g/L, and 20 g/L agar) as per our earlier report [34]. The lipid content of yeast isolates was then measured gravimetrically [43]. More specifically, after cultivation in a lipid production medium at 28 °C for 5 days, the produced yeast biomass was harvested by centrifugation at 12,000 rpm for 10 min. The derived pellet was rinsed three times with distilled water, followed by drying at 105 °C, until constant weight. Quantification of lipids in cell biomass was performed following the method described by Vyas and Chhabra [46]. The lipid content was calculated as a percentage of the lipid weight relative to the dry biomass weight (% w/w). Concurrently, the discharged supernatant was used for glucose and nitrogen determination throughout the cultivation period. The 3,5-Dinitrosalicylic acid method was used to determine residual glucose [99], while nitrogen assimilation was estimated to assess ammonia concentration in the cultivation medium [100].

### Peroxidase production

The selected yeast isolates were tested for MnP production using phenol red as an indicator of ligninolytic activity, with the color of the phenol red changing from deep orange to light yellow [24]. The isolates that showed positive phenol red results were later confirmed for peroxidase production by measuring the development of a reddish-brown color around yeast colonies grown on YEPD agar plates supplemented with the peroxidase substrate (o-Dianisidine dihydrochloride, 0.5 g/L) upon the addition of 0.4 mM H_2_O_2_. Subsequently, the selected isolates were incubated in Bushnell Hass medium containing 0.5 g/L o-Dianisidine dihydrochloride and 150 µM of MnSO_4_.4H_2_O as an inducer to stimulate MnP production. MnP activity in a liquid medium was also determined following Orth et al. [101]. The MnP-producing oleaginous yeasts were then tested for dye decolorization using Acid Orange 7 (AO7) as a model azo dye.

### Azo dye decolorization and enrichment

Decolorization experiments were carried out in Bushnell Hass broth medium amended with AO7 dye at an initial concentration of 50 mg/L and 150 µM of MnSO_4_.4H_2_O as an inducer to stimulate MnP production. Aliquots (10%, v/v) of MnP-producing oleaginous yeast cultures (OD_600_ of 0.2) were inoculated into flasks, which were incubated at 28 °C under static conditions. When the decolorization was realized, a 10 mL mixed culture sample was inoculated into a new dye-added medium for another round of enrichment. The procedures were repeated ten times until the decolorization efficiency was stable. An inoculated dye-free medium was used as an abiotic control. The dye decolorization ability of the enriched isolates was also evaluated on agar plates containing 50 mg dye/L. The fastest-growing MnP-producing oleaginous yeast colonies capable of azo dye decolorization and exhibiting a high ratio of a zone of decolorization to a colony diameter were selected for identification and further experiments. In addition, the performance of the constructed yeast consortium on the decolorization of various textile azo dyes was studied.

Dye decolorization was mointored quantitatively at its corresponding maximum wavelength (Fig. 9) using a UV–Vis spectrophotometer [81, 82]. The culture supernatant was centrifuged (10,000 xg, 10 min, 4 °C) and the percentage of decolorization (%D) was calculated following the formula given by Ali et al. [35]. All assays were carried out in triplicate, with the average values used in calculations. %D = (A_0_ − A_t_)/A_0_ ∗ 100, where A_0_ and A_t_ are the absorbances of dye solutions before and after decolorization, respectively.

The simulated wastewater was prepared according to the real textile dyeing effluent, including mixture of nine azo dyes (Fig. 9) at a concentration of 150 mg/L for each dye. The prepared medium was then supplemented with the synthetic YME substrate to obtain a final concentration of 250 mg/L. The performance of the constructed yeast consortium on the decolorization of the simulated wastewater evaluated using UV–Vis spectrophotometric analysis in the range of 400–800 nm.

### Molecular identification and phylogenetic analysis

The genomic DNA of the selected MnP-producing oleaginous yeast isolates capable of azo dye decolorization was extracted using Dr. GenTLER High Recovery (TakaRa, Japan) according to the manufacturer's instructions. For the D1/D2 and ITS regions, the isolated DNA was amplified with NL1/NL4 and ITS1/ITS4 primers, respectively [36]. The PCR amplification was performed as previously described [32] and the amplification products were sequenced at Sangon Biotech (Shanghai, China). The yeast strains were identified using the nucleotide BLAST (http://www.ncbi.nlm.nih.gov/BLAST/) database. Molecular evolutionary genetics analysis version 7.0 (MEGA 7.0) software was used for phylogenetic and evolutionary analyses [102].

### Construction of MnP-producing oleaginous yeast consortium capable of azo dye decolorization

The NYC-1 consortium stands for molecularly identified MnP-producing oleaginous yeast species *caribbica*, *hansenii*, *guilliermondii*, and *humicola* were developed. A loopful of each yeast strain was cultured separately in screw cap tubes containing 5 mL YEPD broth and incubated at 28 °C for up to 48 h for inoculum preparation. The obtained culture biomass (1.0 g wet weight) was washed three times with phosphate buffer (pH 7.4) before being aseptically inoculated into 100 mL of the same buffer (50 mM) and gently mixed to prepare a homogeneous cell suspension. Finally, the NYC-1 consortium was developed by mixing the cell suspensions at a 1:1 rate.

### Enzyme assays

Activities of MnP, lipase, β-glucosidase, CMCase, and xylanase enzymes produced by the individual strains (SSA1547, SSA1514, SSA1654, and SSA1502) consisting the NYC-1 yeast consortium were determined spectrophotometrically in cell-free extracts. The MnP activity of yeast strains was determined following the method described by Rekik et al. [103]. After adding H_2_O_2_ to the reaction mixture, the increase in the absorbance at 510 nm was monitored for 2 min (30 s interval) at 40 °C. Lipase activity was determined using *p*-nitrophenyl palmitate as a substrate [104]. On the basis of the 3,5-Dinitrosalicylic acid technique [99], the activities of endo-β-1,4-glucanase (CMCase) and xylanase were assessed using CMC and xylan as corresponding substrates, respectively [47]. β-glucosidase enzymatic activity was measured using 5 mM *p*-nitrophenyl glucopyranoside (pH 5) as the corresponding substrate [34]. One unit of enzyme activity was defined as the amount of enzyme required to release 1.0 μmol of the reaction product per minute under specified conditions.

### Effect of lignocellulose degradation inhibitors on yeast growth and lipid accumulation

The effect of the predominant lignocellulose degradation inhibitors (furfural, 5-hydroxymethyl furfural, acetic acid, vanillin, and formic acid) as common inhibitors in lignocellulosic hydrolysates was studied on growth and lipid accumulation. The effect of these inhibitors on growth of the selected individual strains (SSA1547, SSA1514, SSA1654, and SSA1502) consisting the NYC-1 yeast consortium was investigated as described previously [23, 55]. The consortium members' ability to grow in the presence of furfural (0.5, 1.0 and 1.5 g/L), 5-hydroxymethyl furfural (0.5, 1.0, 2.0, and 2.5 g/L), acetic acid (0.5, 1.0, 1.5, and 2.5 g/L), vanillin (0.5, 1.0, 2.0, and 2.5 g/L), and formic acid (0.5, 1.0, 3.0, and 4.0 g/L), which are commonly found in lignocellulosic hydrolysate [55, 69, 105], was tested in Erlenmeyer flasks with yeast nitrogen base medium supplemented with 1% glucose [23] against the control (medium without addition of any inhibitor compound). An aliquot (20 µL) of each yeast cell suspension (10^8^ cells/mL) was inoculated in the prepared sterilized media. Growth was estimated within 14 days of incubation in a rotary shaker at 28 °C with an agitation speed of 150 rpm. Growth was defined following Poontawee et al. [106].

The effect of lignocellulose degradation inhibitors on lipid accumulation by the selected individual strains consisting the NYC-1 consortium was also studied. The prepared inoculum of yeast cultures was inoculated in 50 mL of nitrogen base medium broth supplemented with 1% glucose and in the presence of various concentrations of lignocellulose degradation inhibitors, giving an initial cell concentration of 1.0 at OD_600_. The inoculated flasks were incubated in a rotary shaker at 28 °C with an agitation speed of 150 rpm for 7 days. Cells were subsequently harvested for determining biomass and lipid accumulation. In this experiment, various concentrations of lignocellulose degradation inhibitors were used, including furfural (0.05, 0.1, 0.2 and 0.5 g/L), 5-hydroxymethyl furfural (0.1, 0.5, 1.0, and 5.0 g/L), acetic acid (0.1, 0.5, and 1.0 g/L), vanillin (0.1, 0.5, and 1.0 g/L), and formic acid (0.1, 0.2, 0.3, and 0.5 g/L). The control was a medium without the addition of any inhibitor compound.

### Lipid extraction and biodiesel properties

TLC with a K6 silica gel plate (Merck, India) and a solvent mixture of hexane: diethyl-ether: methanol: acetic acid (78:17:3:2, v/v) were employed. Lipid analysis was detected following exposure to iodine vapor [107]. The microbial lipid generated after methanolysis was examined following Morrison and Smith [108]. The FAME obtained by transesterification was further analyzed as per our earlier report [23]. The main qualitative physicochemical characteristics of the biodiesel produced by the lipid-derived from *Meyerozyma caribbica* and the NYC-1 consortium were determined, including cetane number, kinematic viscosity, iodine, saponification value, oxidative stability, density, long-chain saturation factor, and unsaturation degree [92]. Subsequently, a comparison with palm oil [94] and the oleaginous yeast *Rhodotorula glutinis* R4 [93] was made, followed by an emphasis on the specifications described in the international biodiesel standards, EN 14214 (Europe) and ASTM D6751-3 (USA).

### Statistical analysis

All experiments were carried out in triplicate, and the results were analyzed using NCSS 2020 (LIC, Utah, USA) and Minitab version 19.2020.1 (Minitab Inc., US). The values represent the mean of three independent replicates, with error bars indicating the standard deviation. In terms of statistical significance evaluation, one-way analysis of variance (ANOVA) with Tukey–Kramer multiple comparisons and *t*-Student's tests, applied at *p* value ≤ 0.05.

## Supplementary Information

**Additional file 1: Table S1. **Fatty acid composition of *M. caribbica* SSA1654. **Fig. S1.** UV–Vis spectrophotometric analysis at 400–800 nm of the simulated wastewater containing 250 mg/L of each applied dye, within 21 h of incubation in the presence of the NYC-1 consortium.

## Data Availability

The data sets used and/or analyzed during the current study are available from the corresponding author on reasonable request.
