# Supplementary material for: Wood-feeding termite gut symbionts as an obscure yet promising source of novel manganese peroxidase-producing oleaginous yeasts intended for azo dye decolorization and biodiesel production
Source: Biotechnol Biofuels. 2021 Dec 4;14:229. doi: 10.1186/s13068-021-02080-z (PMC8645103; doi:10.1186/s13068-021-02080-z)
Supplement: Supplementary file 1 — Additional file 1: Table S1. Fatty acid composition of M. caribbica SSA1654. Fig. S1. UV–Vis spectrophotometric analysis at 400–800 nm of the simulated wastewater containing 250 mg/L of each applied dye, within 21 h of incubation in the presence of the NYC-1 consortium. [file 13068_2021_2080_MOESM1_ESM.docx]

**Additional file 1**

**Wood‑feeding termite gut symbionts as an obscure yet promising source** **of novel manganese peroxidase-producing oleaginous yeasts intended for azo dye decolorization and biodiesel production**

Rania Al-Tohamy^a^, Jianzhong Sun^a,*^, Maha A. Khalil^b^, Michael Kornaros^c,d^, Sameh Samir Ali^a,e,*^

^a^Biofuels Institute, School of the Environment and Safety Engineering, Jiangsu University, Zhenjiang, 212013, China

^b^Department of Biology, College of Science, Taif University, P.O. Box 11099, Taif 21944, Saudi Arabia

^c^Laboratory of Biochemical Engineering & Environmental Technology (LBEET), Department of Chemical Engineering, University of Patras, 1 Karatheodori Str., University Campus, 26504 Patras, Greece

^d^INVALOR: Research Infrastructure for Waste Valorization and Sustainable Management, University Campus, 26504 Patras, Greece

^e^Botany Department, Faculty of Science, Tanta University, Tanta, 31527, Egypt

*Corresponding authors at Biofuels Institute, School of the Environment and Safety Engineering, Jiangsu University, Xuefu Rd. 301, 212013, Zhenjiang, China.

**E-mail**: [jzsun1002@ujs.edu.cn](mailto:jzsun1002@ujs.edu.cn) (J. Sun);

[samh@ujs.edu.cn](mailto:samh@ujs.edu.cn), [samh_samir@science.tanta.edu.eg](mailto:samh_samir@science.tanta.edu.eg) (S.S. Ali)

**Table S1.** Fatty acid composition of *M. caribbica* SSA1654.

| Relative fatty acid content (%, w/w) | | |
| --- | --- | --- |
|  | SSA1654 strain | Palm oil |
| Myristic acid (C14:0) | 1.78 | 0.7 |
| Pentadecanoic acid (C15:0) | 0.59 | ND |
| Palmitic acid (C16:0) | 17.34 | 36.7 |
| Palmitoleic acid (C16:1) | 1.60 | 0.1 |
| Stearic acid (C18:0) | 1.86 | 6.6 |
| Oleic acid (C18:1) | 60.73 | 46.1 |
| Linoleic acid (C18:2) | 9.56 | 8.6 |
| Linolenic acid (C18:3) | 4.07 | 0.3 |
| Total C16 | 18.94 | 36.8 |
| Total C18 | 76.22 | 61.6 |
| Total SFA | 19.2 | 43.3 |
| Total MUFA | 62.33 | 46.2 |
| Total PUFA | 13.63 | 8.9 |
| Reference | This study | Ramos et al. [94] |

*Meyerozyma caribbica* SSA1654 was grown for 120 h in the N-limited medium supplemented with 40 g/L of glucose. The values are mean of three independent experiments. **ND**, not determined; **SFA**, saturated fatty acid, **MUFA**, mono-unsaturated fatty acid, and **PUFA**, poly-unsaturated fatty acid.


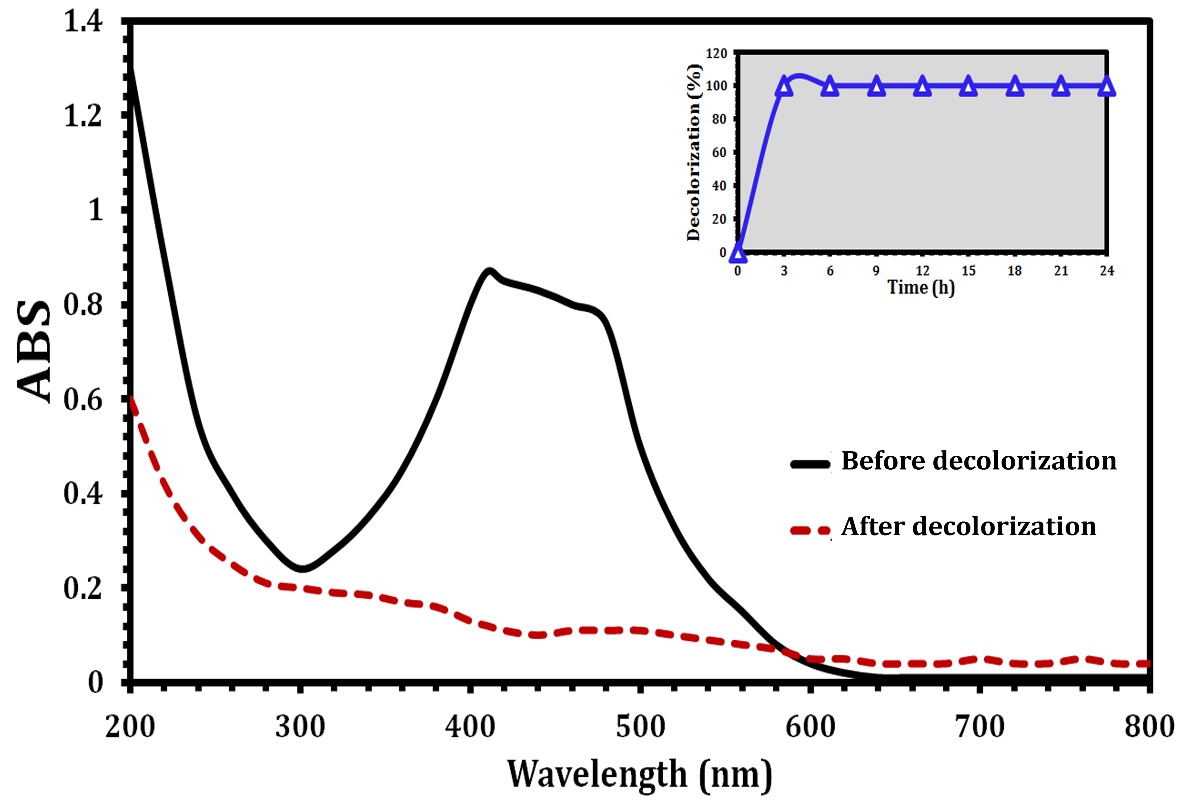


**Fig. S1** UV–vis spectrophotometric analysis at 400-800 nm of the simulated wastewater containing 250 mg/L of each applied dye, within 21 h of incubation in the presence of the NYC-1 consortium.
